# AGTR1 signaling contributes to tumor immunity and therapy response in non-small cell lung cancer

**DOI:** 10.1016/j.omton.2026.201253

**Published:** 2026-05-29

**Authors:** Tatsuki Ikoma, Keigo Araki, Mai Kitagawa, Natsuno Makihara, Yutaro Nagata, Kazuki Fujii, Yukiko Okuno, Keisuke Kamisako, Yuta Okazaki, Kentaro Nakanishi, Yume Sanada, Kiyori Yoshida, Kahori Nakahama, Yuki Takeyasu, Utae Katsushima, Yuta Yamanaka, Satoshi Ikeda, Hiroshige Yoshioka, Toshio Shimizu, Takayasu Kurata

**Affiliations:** 1 Department of Thoracic Oncology, Kansai Medical University, 2-5-1, Shinmachi, Hirakata-city, Osaka 573-1010, Japan; 2 Department of New Experimental Therapeutics, Kansai Medical University Hospital, 2-3-1, Shinmachi, Hirakata-city, Osaka 573-1191, Japan; 3 Department of Clinical Oncology, Kansai Medical University Hospital, 2-3-1, Shinmachi, Hirakata-city, Osaka 573-1191, Japan; 4 Department of Respiratory Medicine and Clinical Immunology, Dokkyo Medical University Saitama Medical Center, Saitama, Japan

**Keywords:** immune checkpoint blockade, ICB, cancer-associated fibroblasts, CAFs, angiotensin II receptor type 1, AGTR1, TGF-β, transforming growth factor β, angiotensin receptor blocker, ARB

## Abstract

Angiotensin II receptor type 1 (*AGTR1*) has emerged as a potential modulator of the tumor microenvironment, yet its role in the immunotherapy response in non-small cell lung cancer (NSCLC) remains unclear. We performed a comprehensive analysis using bulk RNA sequencing and single-cell RNA sequencing (scRNA-seq) datasets. Functional validation included IHC for *AGTR1* and phospho-SMAD2/3 co-localization. The clinical impact of angiotensin receptor blocker (ARB) use was assessed in patients receiving immune checkpoint blockade (ICB) alone or combined with chemo-immunotherapy. *AGTR1*-high expression in NSCLC was associated with pronounced pathway modulation, including significant upregulation of TGF-β, etc. scRNA-seq analysis revealed that *AGTR1* was predominantly localized in cancer-associated fibroblasts (CAFs). IHC validation in NSCLC specimens (*n* = 14) demonstrated a strong correlation between stromal AGTR1 and phospho-SMAD2/3 expression and ARB-treated patients showed significantly reduced stromal *AGTR1* and pSMAD2/3 expression. Clinically, ARB treatment showed no benefit in ICB monotherapy but significantly improved PFS in chemo-immunotherapy (HR = 0.70, *p* = 0.01), especially in non-squamous (non-Sq) histology with PD-L1≥1% (HR = 0.52, *p* = 0.01). AGTR1 is predominantly expressed in CAFs and is suggestive of a correlation with TGF-β-related immunosuppressive features in NSCLC. The combination of ARB with chemo-immunotherapy may enhance therapeutic efficacy through targeting that axis in CAFs, specifically in non-Sq NSCLC patients.

## Graphical abstract

**Figure:**
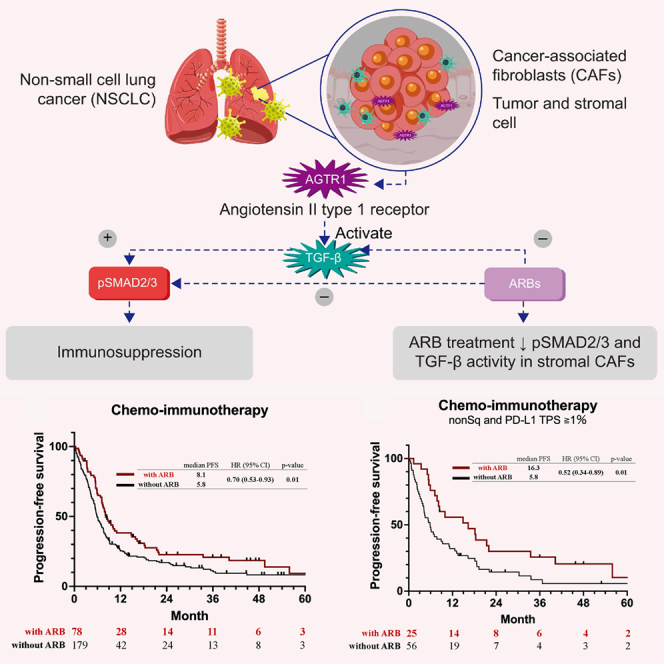

## Teaser

Ikoma and colleagues show that AGTR1 is predominantly expressed in cancer-associated fibroblasts and is associated with activation of TGF-β signaling in NSCLC. In a retrospective analysis, concomitant angiotensin receptor blocker use during chemo-immunotherapy was significantly associated with improved progression-free survival in non-squamous NSCLC, suggesting ARBs as a potential stromal-targeting strategy.

## Introduction

Immune checkpoint blockade (ICB) possesses the ability to activate patients’ endogenous T cells, thereby promoting tumor elimination.^1^ These therapeutic agents, initially introduced for melanoma treatment, have now established themselves as a cornerstone of standard care across numerous cancer types.^1^ Furthermore, the advent of ICBs has led to an increase in cases achieving long-term survival, undoubtedly establishing these agents as revolutionary therapeutics in cancer treatment. However, not all patients respond to these drugs. Unlike conventional therapies, ICBs represent a unique class of agents in that even within the same cancer type, there exists a dichotomy between responder and non-responder populations.

The tumor microenvironment (TME), which surrounds the cancer, has gained attention as a key determinant of this heterogeneity in treatment response among homogeneous cancer populations.^2^ The TME includes complex components, such as immune cells, including T cells and diverse stromal cells including fibroblasts.^3^ These stromal cells have been shown to suppress tumor immunity, leading to limited anticancer effects when treated with not only ICB monotherapy but also chemo-immunotherapy.^4^^,^^5^ Fibroblasts, especially cancer-associated fibroblasts (CAFs), produce collagen fibers in the peritumoral region, promoting fibrosis progression and creating an environment that hinders immune cell infiltration. Therefore, overcoming fibrosis may represent a crucial step toward successful immunotherapy.

Non-small cell lung cancer (NSCLC) is one of the malignancies for which immunotherapy has become standard treatment.^6^ As mentioned above, numerous cases have demonstrated dramatically improved prognosis following the advent of immunotherapy. However, there also exists a population that remains refractory to immunotherapy with persistently poor prognosis. In NSCLC, CAFs have been reported to correlate with prognosis and treatment efficacy, making the targeting of CAFs an urgent challenge.^7^ Angiotensin II receptor 1 (*AGTR1*) has been identified in multiple reports as a potential target for CAF regulation.^8^^,^^9^^,^^10^ Angiotensin receptor blockers (ARBs) are agents that inhibit the *AGTR1* receptor. ARBs were initially developed as antihypertensive drugs and are widely prescribed medications. Recent studies have explored their potential for drug repositioning in cancer treatment beyond their use as antihypertensives.^10^^,^^11^ However, most investigations remain limited to preclinical data, and studies examining the impact of ARBs on antitumor effects in first-line immunotherapy for NSCLC remain scarce. Therefore, we investigated the role of *AGTR1* in tumor and stromal cells, as well as its impact on treatment effectiveness in NSCLC, utilizing both public databases and institutional data.

## Results

### AGTR1 expression demonstrates distinct pathway modulation in lung adenocarcinoma

**Figure 1:**
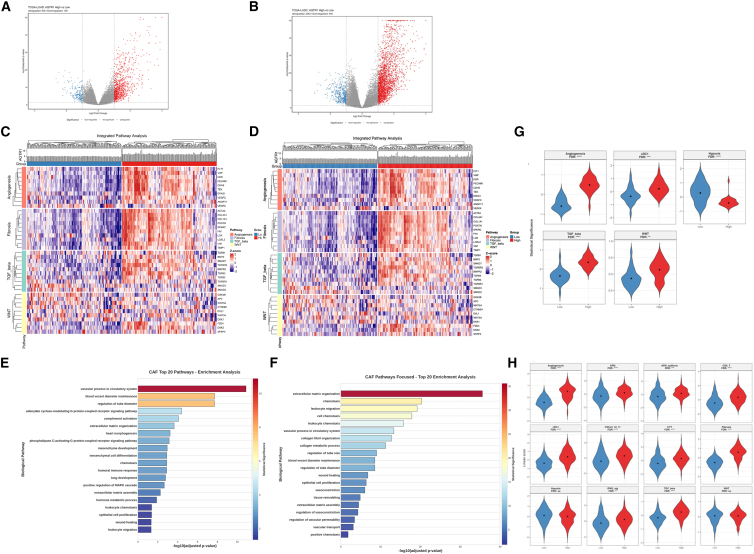
*AGTR1* expression analysis and pathway enrichment in lung cancer datasets (A and B) Volcano plots showing differentially expressed genes between *AGTR1*-high and *AGTR1*-low groups in TCGA-LUAD (A) and TCGA-LUSC (B). Red dots indicate upregulated genes, blue dots indicate downregulated genes, and gray dots indicate non-significant genes. Vertical dashed lines represent log2 fold change thresholds (±1), and the horizontal dashed line represents the adjusted *p* value threshold (0.05). (C and D) Heatmaps displaying integrated pathway, including angiogenesis, fibrosis, TGF-beta, and WNT/b-catenin, analysis of differentially expressed genes in LUAD (C) and LUSC (D). Samples are grouped by AGTR1 expression levels (low vs. high). The color scale represents pathway activity scores (*Z*-scores), with red indicating high activity and blue indicating low activity. (E and F) Bar plots showing Gene Ontology enrichment analysis of biological pathways in LUAD (E) and LUSC (F). The *x* axis represents—log10 (adjusted *p* value), and the *y* axis lists enriched biological processes. The color gradient indicates statistical significance, with red indicating higher significance. Key enriched pathways include vascular processes, extracellular matrix organization, and chemotaxis. (G and H) Violin plots comparing pathway signature scores between *AGTR1*-low (blue) and *AGTR1*-high (red) groups in LUAD (G) and LUSC (H). Multiple pathway signatures are displayed, including Angiogenesis, conventional dendritic cell 1 (cDC1), Hypoxia, TGF-beta, and WNT/b-catenin pathways. Black dots represent median values, and error bars indicate interquartile ranges. Statistical significance is denoted as: ∗*p* < 0.05, ∗∗*p* < 0.01, ∗∗∗*p* < 0.001, ∗∗∗∗*p* < 0.0001. FDR, false discovery rate.

Bulk RNA sequencing (RNA-seq) analysis revealed that, regardless of histological subtype (LUAD or lung squamous cell carcinoma, LUSC), *AGTR1*-high expression groups showed differential expression of diverse genes compared to low-expression groups (Figures 1A and 1B). A total of 1,096 and 2,809 DEGs were identified in LUAD and LUSC, respectively, of which 558 (16.7%) were shared between the two subtypes, with 95.7% showing concordant direction of change. In LUAD, the top upregulated DEGs besides *AGTR1* included *SLC6A4*, *INMT*, *ADAMTS7P3*, *GPIHBP1*, *TCF21*, *VEGFD*, *AGER*, *PRG4*, *ADH1B*, and *ABCA8*, whereas in LUSC, *SPON1*, *COLEC12*, *TCF21*, *DLC1*, *ZCCHC24*, *CRISPLD2*, *AOC3*, *JAM2*, *DAAM2*, and *SNED1* were among the top upregulated genes. Notably, *TCF21*, a known fibroblast marker, was shared between both subtypes. Heatmap analysis also revealed distinct patterns in gene expression between the two groups for genes associated with representative pathways, including angiogenesis, fibrosis, WNT/β-catenin signaling, and transforming growth factor β (TGF-β) signaling (Figures 1C and 1D).

Furthermore, gene set enrichment analysis (GSEA) analysis revealed that pathways involved in angiogenesis, extracellular matrix (ECM), and fibrosis were significantly enriched in the *AGTR1*-high expression group relative to the *AGTR1*-low group (Figures 1E and 1F). We performed pathway signature analyses comparing *AGTR1*-high versus *AGTR1*-low expression groups across three cohorts (The Cancer Genome Atlas [TCGA]-LUAD, TCGA-LUSC, and an independent adenocarcinoma cohort GSE32863) (Figures 1G and 1H; Figure S1F). The LUAD dataset demonstrated the most pronounced pathway modulation, with *AGTR1*-high tumors showing significant upregulation of angiogenesis (diff = 0.45, adjusted *p* = 3.36 × 10^−12^), TGF-β (diff = 0.25, adjusted *p* = 9.14 × 10^−6^), hypoxia, and the conventional dendritic cell 1 (cDC1) signature, all of which exhibited distinct bimodal distributions with minimal overlap between groups. The independent adenocarcinoma cohort corroborated these findings, with similar upregulation of angiogenesis, TGF-β, cytolytic activity, and cDC1, though it notably showed a decreased hypoxia signature.

In contrast, the LUSC dataset exhibited a distinct pattern of pathway activation. While a broader range of pathways reached statistical significance, including APM (diff = 0.49, adjusted *p* = 9.53 × 10^−6^), CD8+ T cells (diff = 0.74, adjusted *p* = 4.46 × 10^−11^), CXCL9/10/11 (diff = 0.52, adjusted *p* = 9.44 × 10^−6^), cytolytic activity, and fibrosis (diff = 1.11, adjusted *p* = 2.64 × 10^−36^), these immune-related signatures were not significantly associated with *AGTR1* expression in the LUAD dataset. These findings suggest that *AGTR1* expression in LUAD is predominantly linked to TGF-β- dependent stromal remodeling, whereas in LUSC, *AGTR1* correlates with a more diverse microenvironmental profile encompassing both stromal and immune compartments. Across all cohorts, angiogenesis and TGF-β signatures were consistently upregulated, and cDC1 levels were elevated in all cohorts.

### AGTR1 is predominantly expressed in CAFs and regulates TME remodeling

**Figure 2:**
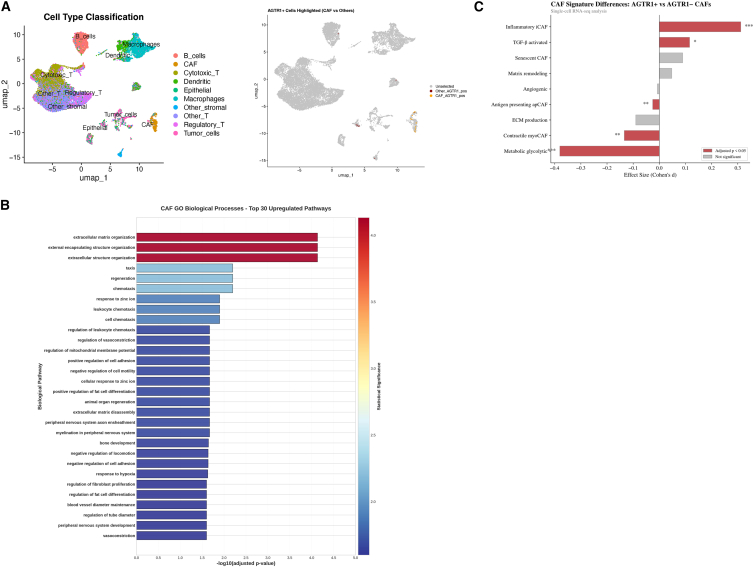
Single-cell RNA sequencing analysis reveals *AGTR1* expression predominantly in cancer-associated fibroblasts (A) Left: UMAP plot showing cell type classification in lung cancer tissue. Eleven distinct cell populations are identified, including B cells, CAF, cytotoxic T cells, dendritic cells, epithelial cells, macrophages, other stromal cells, other T cells, regulatory T cells, and tumor cells. Right: UMAP plot highlighting *AGTR1*-positive cells, with orange dots representing CAF cells expressing *AGTR1* and red dots representing other cell types expressing *AGTR1*. Gray dots indicate *AGTR1*-negative cells. (B) Horizontal bar plot showing GO enrichment analysis of biological pathways in the single-cell dataset. The *x* axis represents −log10(adjusted *p* value), and the *y* axis lists enriched biological processes. The color gradient indicates statistical significance, with red indicating the highest importance. Top enriched pathways include extracellular matrix organization, external encapsulating structure organization, and various chemotaxis-related processes. (C) Bar plot showing effect sizes (Cohen’s d) of CAF-related signature scores comparing *AGTR1*-positive and *AGTR1*-negative CAFs from single-cell RNA-seq analysis.

To determine the cellular localization of *AGTR1* in the lung adenocarcinoma microenvironment, we analyzed two independent single-cell RNA sequencing (scRNA-seq) datasets. Uniform manifold approximation and projection (UMAP) visualization demonstrated that *AGTR1* expression was predominantly localized to CAFs, with minimal expression detected in a small subset of tumor cells (Figure 2A).

To elucidate the functional role of *AGTR1* in CAFs, we compared pathway enrichment between *AGTR1*-positive and *AGTR1*-negative CAF populations. Bar plot analysis revealed that *AGTR1*+ CAFs exhibited significant enrichment of multiple pathways involved in microenvironment remodeling (Figure 2B). Especially, scRNA-seq analysis revealed that the TGF-β signaling pathway was robustly upregulated in *AGTR1*+ CAFs (Figure 2C). *AGTR1*+ CAFs exhibited significantly elevated TGF-β activated signature scores (mean difference = 0.026, effect size = 0.116, adjusted *p* value = 0.037). However, statistical significance was not achieved across all pathways. In contrast, a comparative analysis of *AGTR1*+ versus *AGTR1*-tumor cells across both datasets revealed no consistent pattern of pathway enrichment, suggesting that the stromal compartment primarily drives *AGTR1*-mediated effects on the microenvironment.

### IHC analysis of *AGTR1* and TGF-β pathway activation

Our bioinformatics analysis of publicly available datasets suggested an association between *AGTR1* expression and activity of the TGF-β signaling pathway. To further explore this association in clinical specimens, we performed immunohistochemical (IHC) analysis using pathological specimens from a subset of non-squamous NSCLC patients treated at our institution (*n* = 14; ARB-treated, *n* = 5; non-ARB-treated, *n* = 9).

**Figure 3:**
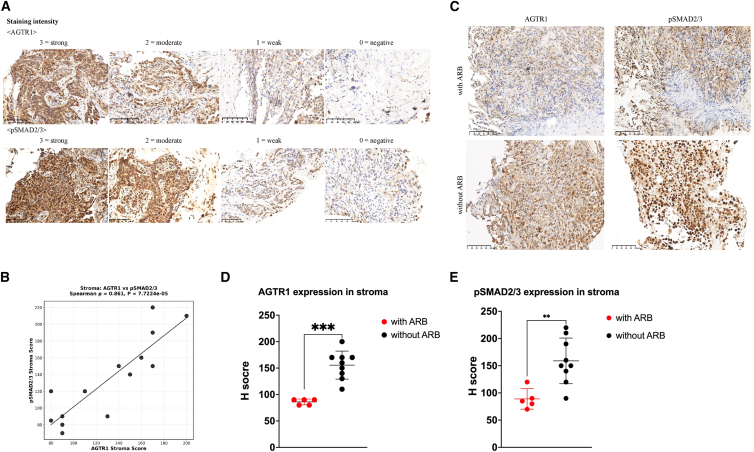
Immunohistochemical analysis of *AGTR1* and pSMAD2/3 expression in lung cancer tissues and correlation with ARB treatment (A) Representative immunohistochemical images showing *AGTR1* and pSMAD2/3 staining intensity in lung cancer stromal cells. Staining intensity is scored as: 3 = strong, 2 = moderate, 1 = weak, 0 = negative. Scale bars, 100 μm. (B) The scatterplot shows a positive correlation between *AGTR1* and pSMAD2/3 expression in stromal cells (*n* = 14 patients). The Spearman’s correlation coefficient is *ρ* = 0.861, and the *p* value is 7.7224e−05. Each dot represents an individual patient sample, and the line shows the linear regression fit. (C) Representative immunohistochemical images comparing *AGTR1* (left) and pSMAD2/3 (right) expression in stromal cells between patients with ARB treatment (top) and without ARB treatment (bottom). (D) Scatterplot showing *AGTR1* expression levels (H-score) in stromal cells. Red dots represent patients with ARB treatment (*n* = 5), and black dots represent patients without ARB treatment (*n* = 9). Horizontal lines indicate mean ± standard deviation. *AGTR1* expression is significantly lower in the ARB group compared to the non-ARB group (∗∗∗*p* < 0.001, Mann-Whitney *U* test). (E) Scatterplot showing pSMAD2/3 expression levels (H-score) in stromal cells. Red dots represent patients with ARB treatment (*n* = 5), and black dots represent patients without ARB treatment (*n* = 9). Horizontal lines indicate mean ± standard deviation. pSMAD2/3 expression is significantly lower in the ARB group compared to the non-ARB group (∗∗*p* < 0.01, Mann-Whitney *U* test).

We evaluated *AGTR1* and phosphorylated SMAD2/3 (pSMAD2/3), a marker of TGF-β pathway activation, by IHC (Figure 3A). *AGTR1* and pSMAD2/3 were stained on separate slides prepared from the same formalin-fixed paraffin-embedded (FFPE) tumor blocks of each patient; multiplex co-staining was not performed in this study. The staining intensity was assessed using a four-tier scoring system as illustrated in the figures. Based on our previous analyses indicating predominant *AGTR1* expression in CAFs, we focused our evaluation on the tumor stromal compartment.

In the stromal regions, we observed a significant positive correlation between *AGTR1* expression and pSMAD2/3 expression (Spearman’s *ρ* = 0.86, *p* = 7.22 × 10^−5^; Figure 3B). Representative histological images are shown in Figure 3C. Notably, patients who received ARB treatment exhibited significantly lower stromal *AGTR1* expression than those without ARB treatment (H-score: 86.0 ± 5.4 vs. 155.6 ± 26.5, *p* value <0.001; Figure 3D). Furthermore, pSMAD2/3 expression was also considerably reduced in ARB-treated patients (H-score: 89.0 ± 18.8 vs. 158.9 ± 41.9, *p* value <0.01; Figure 3E), consistent with a correlative association between stromal *AGTR1* expression and TGF-β pathway activity in the TME.

### Clinical outcomes and impact of ARB treatment on the effectiveness of immunotherapy

**Figure 4:**
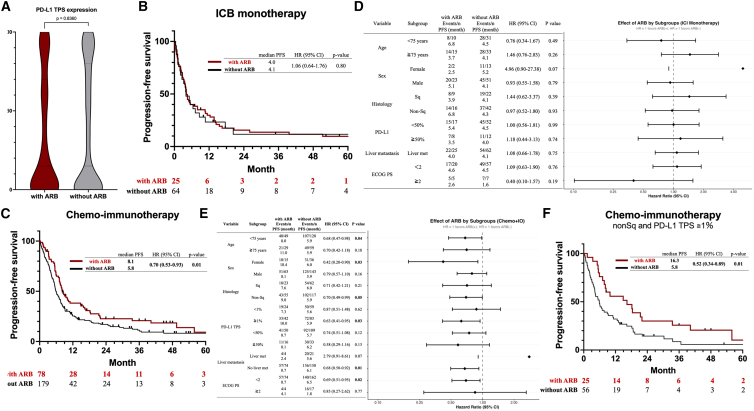
Clinical outcomes and progression-free survival analysis in lung cancer patients stratified by ARB treatment (A) Violin plot comparing PD-L1 TPS distribution between patients with ARB treatment (red, *n* = 91) and without ARB treatment (black, *n* = 206) in the entire study cohort. The width of each violin represents the density of data points at different values. Black dots indicate median values, and error bars represent interquartile ranges. No significant difference was observed between the two groups (33.7 ± 37.2% vs. 37.0 ± 39.1%, *p* = 0.83, Mann-Whitney U test). (B) Kaplan-Meier curve showing PFS in patients treated with ICB monotherapy. The red line represents patients with ARB treatment (*n* = 25), and the black line represents patients without ARB treatment (*n* = 64). Median PFS: 4.0 months (with ARB) vs. 4.1 month (without ARB); HR = 1.06 (95% CI 0.64–1.76), *p* = 0.80 (log rank test). (C) Kaplan-Meier curve showing PFS in patients treated with chemo-immunotherapy. The red line represents patients with ARB treatment (*n* = 78), and the black line represents patients without ARB treatment (*n* = 179). Median PFS: 8.1 months (with ARB) vs. 5.8 months (without ARB); HR = 0.70 (95% CI 0.53–0.93), *p* = 0.01 (log rank test). (D) Forest plot showing subgroup analysis of ARB effect on PFS in patients receiving ICB monotherapy. Variables analyzed include age, sex, histology, PD-L1 expression, liver metastasis, and ECOG PS. Hazard ratios with 95% confidence intervals are displayed. No subgroup showed a significant benefit from ARB treatment. (E) Forest plot showing subgroup analysis of ARB effect on PFS in patients receiving chemo-immunotherapy. Variables analyzed include age, sex, histology, PD-L1 TPS, liver metastasis, and ECOG PS. Several subgroups showed significant benefit from ARB treatment, particularly in non-Sq histology (*p* = 0.05), PD-L1 TPS ≥1% (*p* = 0.03). (F) Kaplan-Meier curve showing PFS in chemo-immunotherapy patients with non-squamous histology and PD-L1 TPS ≥1%. The red line represents patients with ARB treatment (*n* = 25), and the black line represents patients without ARB treatment (*n* = 56). Median PFS: 16.3 months (with ARB) vs. 5.8 months (without ARB); HR = 0.52 (95% CI 0.34–0.89), *p* = 0.01.

We evaluated the impact of ARB treatment on clinical outcomes using data from patients treated at our institution. First, we assessed whether ARB administration influenced PD-L1 tumor proportion score (TPS) expression. No significant difference in PD-L1 TPS was observed between ARB-treated and non-treated patients (33.7% ± 37.2% vs. 37.0% ± 39.1%, *p* value = 0.83), indicating that ARB does not directly modulate tumor PD-L1 expression (Figure 4A).

Patient baseline characteristics for the ICB monotherapy (*n* = 89) and chemo-immunotherapy (*n* = 207) cohorts are summarized in Table 1. The median observation period for this clinical research cohort is 12.5 months (0.19–98.9 months). Within each treatment modality, there were no significant imbalances in baseline characteristics between patients with and without ARB treatment. In the ICB monotherapy group, ARBs included telmisartan (*n* = 7), olmesartan (*n* = 6), valsartan (*n* = 3), and others (*n* = 9). In the chemo-immunotherapy group, the most frequently used agents were candesartan (*n* = 16), telmisartan (*n* = 15), azilsartan (*n* = 10), and others (*n* = 37). In the ICB monotherapy cohort (*n* = 89; ARB *n* = 25, non-ARB *n* = 64), ARB treatment did not confer a substantial benefit in progression-free survival (PFS) (median PFS: 4.0 vs. 4.1 month, hazard ratio (HR, 1.06), 95% confidence interval (CI, 0.64–1.76, *p* value = 0.80; Figure 4B). Subgroup analysis using a forest plot revealed no patient populations that clearly benefited from ARB in this setting (Figure 4D).

**Table 1:** Baseline patient characteristics stratified by ARB treatment in ICB monotherapy and chemo-immunotherapy cohorts

Variable	ICB monotherapy				Chemo-immunotherapy			
	all *n* = 89 (%)	with ARB *n* = 25 (%)	without ARB *n* = 64 (%)	*p* value	all *n* = 257 (%)	with ARB *n* = 78 (%)	without ARB *n* = 179 (%)	*p* value
Age	–	–	–	0.49	–	–	–	0.56
<75	41(46)	10 (40)	31 (48)		169 (66)	49 (63)	120 (67)	
≧75	48 (54)	15 (60)	33 (52)		88 (34)	29 (37)	59 (33)	
Sex	–	–	–	0.21	–	–	–	0.99
Male	74 (83)	23 (92)	51 (80)		206 (80)	63 (81)	143 (80)	
Female	15 (17)	2 (8)	13 (20)		51 (20)	15 (19)	36 (20)	
ECOG PS	–	–	–	0.30	–	–	–	0.32
<2	77 (87)	20 (80)	57 (89)		236 (92)	74 (95)	162 (91)	
≧2	12 (13)	5 (20)	7 (11)		21 (8)	4 (5)	17 (9)	
Tissue	–	–	–	0.99	–	–	–	0.47
Sq	31 (35)	9 (36)	22 (34)		85 (33)	23 (29)	62 (35)	
Non-Sq	58 (65)	16 (64)	42 (66)		172 (67)	55 (71)	117 (65)	
Stage	–	–	–	0.79	–	–	–	0.87
Ⅲ/Ⅳ	63 (71)	17 (68)	46 (72)		187 (73)	56 (72)	131 (73)	
Recurrence	26 (29)	8 (32)	18 (28)		70 (27)	22 (28)	48 (27)	
PD-L1 TPS	–	–	–	0.25	–	–	–	0.76
≧50%	69 (78)	17 (68)	52 (81)		49 (19)	16 (21)	33 (18)	
1∼49%	20 (22)	8 (32)	12 (19)		76 (30)	26 (33)	50 (28)	
<1%	−	−	−		83 (32)	24 (31)	59 (33)	
Unknown	−	−	−		49 (19)	12 (15)	37 (21)	
Metastasis	–	–	–	−	–	–	–	−
Liver	2 (2)	0	2 (3)		25 (10)	4 (5)	21 (12)	
Bone	27 (30)	8 (32)	19 (30)		82 (32)	25 (32)	57 (32)	
Brain	8 (9)	2 (8)	6 (9)		36 (14)	11 (14)	25 (14)	
CTLA-4	–	–	–	−	–	–	–	0.78
Yes	−	−	−		16 (6)	4 (5)	12 (7)	
No	−	−	−		241 (94)	74 (95)	167 (93)	
Bevacizumab	–	–	–	−	–	–	–	0.99
Yes	−	−	−		13 (5)	4 (5)	9 (5)	
No	−	−	−		244 (95)	74 (95)	170 (95)	

ARB, angiotensin receptor blocker; ECOG PS, Eastern Cooperative Oncology Group performance status; ICB, immune checkpoint blockade; Sq, Squamous cell carcinoma; PD-L1 TPS, PD-L1 tumor proportion score.

In contrast, patients receiving chemo-immunotherapy (*n* = 257; ARB *n* = 78, non-ARB *n* = 179) with concurrent ARB treatment demonstrated significantly prolonged PFS compared to those without ARB (median PFS: 8.1 vs. 5.8 months, HR 0.70, 95% CI 0.53–0.93, *p* value = 0.01; Figure 4C). Subgroup analysis identified specific populations that derived greater benefit from ARB, particularly patients with PD-L1 TPS ≥1% and non-squamous histology (Figure 4E). In the same population, ultivariate Cox regression analysis, adjusting for age, sex, Eastern Cooperative Oncology Group performance status (ECOG PS), PD-L1 TPS, and presence of liver metastasis, confirmed that ARB treatment independently predicted improved PFS in the chemo-immunotherapy cohort (HR 0.52, 95% CI 0.31–0.89, *p* = 0.01; Table 2). When the analysis was restricted to patients with non-squamous histology and PD-L1 TPS ≥1%, the PFS benefit associated with ARB was further prolonged, with median PFS of 16.3 months in the ARB group compared to 5.8 months in the non-ARB group (HR 0.52, 95% CI 0.34–0.89, *p* value = 0.01; Figure 4F). In a complementary analysis, the objective response rate (ORR) was compared between ARB-treated and non-ARB-treated patients. In the ICB monotherapy group, ORR was similar between ARB-treated and non-ARB-treated patients (32.0% vs. 32.8%, *p* > 0.99). In the chemo-immunotherapy group, a numerically higher ORR was observed in ARB-treated patients (62.8% vs. 51.9%), although this difference did not reach statistical significance (*p* = 0.13), likely reflecting the limited sample size of the cohort.

**Table 2:** Univariate and multivariate analysis of progression-free survival in patients with non-squamous histology and PD-L1 TPS ≥1% receiving chemo-immunotherapy

Variable	Chemo-immunotherapy			
	Univariate		Multivariate	
	HR (95% CI)	*p* value	HR (95% CI)	*p* value
**Age**				

≧75	1.06 (0.65-1.75)	0.80	−	−
(ref, <75)				

**Sex**				

Male	1.63 (0.86-3.08)	0.13	−	−
(ref, female)				

**ECOG PS**				

≧2	2.06 (0.97-4.37)	0.05	−	−
(ref, <2)				

**PD-L1 TPS**				

≧50%	1.16 (0.71-1.87)	0.54	−	−
(ref, < 50%)				

**Liver metastasis**				

No	0.62 (0.28-1.36)	0.23	−	−
(ref, Yes)				

**ARB**				

Yes	0.52 (0.31-0.89)	**0.01**	0.52 (0.31-0.89)	**0.01**
(ref, No)				

HR, hazard ratio; CI, confidence interval.

## Discussion

**Figure 5:**
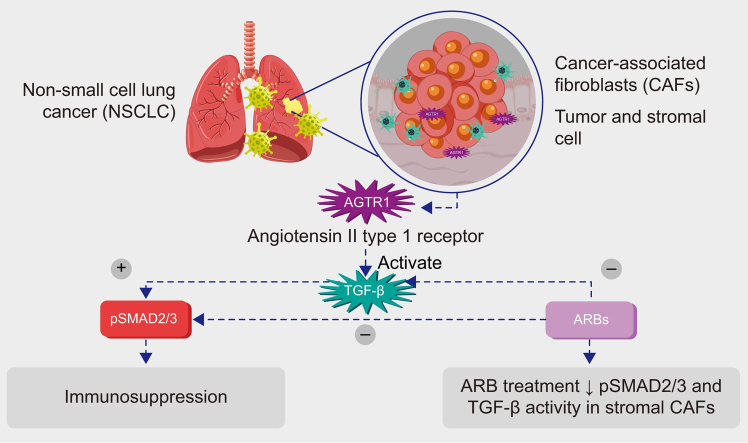
Schematic diagram depicting the regulatory role of *AGTR1* in the TME immune landscape of NSCLC *AGTR1* is predominantly expressed in CAFs, and *AGTR1* expression is suggestive of a correlation with TGF-β-related immunosuppressive features. ARBs, which predominantly inhibit *AGTR1*, may enhance immunotherapy efficacy by modulating CAFs, warranting further therapeutic development in this context.

We performed comprehensive bioinformatic analyses and clinical validation to elucidate the role of *AGTR1* in NSCLC and its therapeutic implications for immunotherapy. Our findings demonstrate that *AGTR1* is predominantly expressed in CAFs and is associated with stromal remodeling in the context of TGF-β signaling activation (Figure 5). Critically, ARB treatment significantly enhanced the effectiveness of chemo-immunotherapy in patients with non-squamous NSCLC and PD-L1 TPS ≥1%, whereas ICB monotherapy provided no benefit.

### CAFs and immunotherapy resistance

CAFs represent critical determinants of immunotherapy resistance.^2^ Based on the present findings, *AGTR1* may constitute a promising molecular target for overcoming CAF-mediated resistance mechanisms to immunotherapy. scRNA-seq and IHC demonstrated that *AGTR1* expression is predominantly localized to the tumor stroma, particularly in CAFs, rather than tumor cells. Of note, the proportion of *AGTR1*-positive CAFs appeared lower in scRNA-seq than in IHC, likely reflecting the known dropout of moderately expressed genes in scRNA-seq and the differences in sensitivity between mRNA and protein detection methods. CAFs not only establish physical barriers that restrict T cell infiltration but also activate multiple signaling pathways that attenuate the efficacy of immunotherapy.^12^ However, consistent with our data, CAFs exhibit substantial heterogeneity in their functional properties.^13^ We identified distinct molecular signatures between *AGTR1*-positive and *AGTR1*-negative CAF subpopulations, particularly in ECM-related pathway activation, indicating that CAFs, analogous to tumor cells, comprise heterogeneous populations with diverse functional states.^14^ This heterogeneity presents significant challenges for pharmacological targeting of all CAF populations. Nevertheless, the identification of specific molecular markers, such as *AGTR1*-expressing CAFs, that contribute adversely to antitumor immunity represents a critical advancement toward the development of targeted therapeutic interventions.

### *AGTR1* expression, TGF-β pathway activity, and therapeutic implications

The inhibitory effect of ARBs on TGF-β signaling is well-established in non-oncological settings, most notably in Marfan syndrome, where losartan attenuates TGF-β-driven aortic remodeling.^15^ In the oncology context, several clinical trials have explored the combination of ARBs with conventional cancer therapies; losartan has been investigated as a stromal-modifying agent in combination with FOLFIRINOX and chemoradiation in locally advanced pancreatic cancer, demonstrating improved R0 resection rates.^16^ Furthermore, retrospective studies have suggested that ARB or ACEI use is associated with improved outcomes in several cancer types, including colorectal and renal cell carcinoma.^17^ However, the role of ARBs in combination with ICB remains largely unexplored. Our results demonstrate that *AGTR1* expression correlated with TGF-β-related transcriptional and IHC features in the TME. Previous investigations have reported that *AGTR1* signaling can promote fibrotic changes and an immunologically “cold” phenotype, findings that are consistent with our observations.^10^^,^^18^ The novelty of the present study lies in demonstrating that *AGTR1* expression in NSCLC is predominantly localized to CAFs, that *AGTR1*+ CAFs showed a correlative association with TGF-β pathway activity, and that concomitant ARB use during chemo-immunotherapy correlates with improved PFS in non-squamous NSCLC patients with PD-L1 TPS ≥1%. These findings provide a TME-specific hypothesis for repurposing ARBs as a candidate stromal-targeting strategy in combination with immunotherapy, warranting further functional and prospective clinical validation. The lower *AGTR1* immunostaining observed in tumors from patients receiving ARB treatment (Figure 3) may reflect long-term disruption of a positive feedback loop in which angiotensin II signaling through *AGTR1* sustains its own transcription. Chronic ARB-mediated receptor blockade may attenuate this autocrine signaling, potentially leading to gradual transcriptional downregulation of *AGTR1* in CAFs. Alternatively, the direct binding of ARBs to *AGTR1* may partially interfere with antibody epitope accessibility, resulting in reduced IHC detection rather than a true decrease in protein expression. These interpretations remain speculative and require functional validation in future studies.

### Differential response patterns in ICB monotherapy and histological subtypes

Analysis of public datasets and clinical cohorts identified two distinct patient subpopulations in which the additive effect of *AGTR1*-targeting ARBs was limited: patients receiving ICB monotherapy and those with squamous cell carcinoma (Sq) patients undergoing chemo-immunotherapy. Regarding ICB monotherapy, our clinical cohort showed no significant improvement in PFS with or without ARB, and subgroup analyses did not reveal any apparent benefit. These findings suggest that *AGTR1* inhibition has been less effective in ICB monotherapy settings. Several mechanisms may explain this observation. First, while ARBs may suppress fibrosis progression, their capacity to reverse established fibrotic architecture may appear limited.^18^ Second, *AGTR1* inhibition may paradoxically activate hypoxia-related pathways. Our data revealed enhanced hypoxia signatures in *AGTR1*-low expression groups. Hypoxia pathway activation promotes immune evasion and the development of cold TMEs, potentially attenuating ICB efficacy.^19^^,^^20^ Third, *AGTR1* inhibition alone is insufficient to induce antigen presentation pathways. Our analysis revealed unchanged or decreased cDC1 and APM-related signatures in *AGTR1*-low groups.^21^ While *AGTR1* inhibition may create a more permissive environment for T cell recruitment, insufficient antigen presentation limits therapeutic improvements. Conversely, the addition of chemotherapy may enhance antigen presentation through immunogenic cell death, enabling fuller exploitation of the benefits of *AGTR1* inhibition.^22^

### Histology-specific mechanisms in squamous cell carcinoma

In Sq patients receiving chemo-immunotherapy, therapeutic benefits remained limited. While angiogenesis- and TGF-β-related pathways were significantly downregulated in *AGTR1*-low Sq tumors, the magnitude of these changes might appear insufficient to translate into clear clinical benefits, particularly when contrasted with LUAD cohorts. Moreover, unlike LUAD, the WNT/β-catenin pathway remained activated in Sq cancers regardless of *AGTR1* expression status, potentially hindering enhancement of T cell-mediated antitumor activity.^23^ These findings collectively suggest that *AGTR1* may serve as a novel therapeutic target to improve the efficacy of chemo-immunotherapy in non-Sq NSCLC.

### Study limitations

This study has several limitations. First, functional analyses of *AGTR1* were primarily based on bioinformatic approaches, and experimental validation—including *AGTR1* knockdown or pharmacologic inhibition in CAFs followed by assessment of pSMAD2/3 and canonical TGF-β target genes—will be required in future studies to formally establish a functional *AGTR1*-TGF-β relationship. Second, the precise molecular mechanisms underlying *AGTR1*-related CAF activation remain to be fully elucidated. Third, this was a single-center retrospective study with limited sample size, requiring prospective clinical trials to confirm therapeutic impact. Fourth, although IHC and transcriptomic analyses consistently indicated an association between *AGTR1* expression and TGF-β signaling activation in CAFs, the present data remain correlative; multiplex co-staining was not performed in this study. Future investigations using multiplex immunofluorescence or spatial transcriptomics to directly demonstrate co-localization of *AGTR1* and pSMAD2/3 at single-cell resolution would be needed to substantiate a spatial relationship between *AGTR1* expression and TGF-β pathway activation.

### Conclusion

This study demonstrates that *AGTR1* represents a poor prognostic factor in lung adenocarcinoma, is predominantly expressed in CAFs, and plays crucial roles in TME remodeling. Therapeutic strategies targeting *AGTR1* signaling may represent novel treatment options for lung adenocarcinoma, particularly in combination with chemo-immunotherapy for non-squamous histology.

## Materials and methods

### Human dataset acquisition

RNA-seq results were obtained, and analyses were conducted using both bulk RNA-seq datasets and scRNA-seq data. Regarding bulk RNA-seq, three datasets were acquired in total: the TCGA lung adenocarcinoma (TCGA-LUAD) and lung squamous cell carcinoma (TCGA-LUSC) datasets, along with the GSE32863 dataset.^24^ For scRNA-seq, two datasets were obtained and analyzed: GSE123902^25^ and GSE253013.^26^

### Bulk transcriptome analysis

Three independent transcriptome datasets were analyzed to investigate *AGTR1* expression and its associated molecular signatures in NSCLC: RNA-seq data from TCGA-LUAD and TCGA-LUSC obtained via TCGAbiolinks, and microarray data from GSE32863 obtained from the Gene Expression Omnibus (GEO) database. Samples were stratified by *AGTR1* expression into quartiles, with Q1 (lowest 25%) and Q4 (highest 25%) selected for comparative analysis. Differential expression analysis was performed using DESeq2 for RNA-seq data and limma for microarray data. DEGs were defined as adjusted *p* value <0.05 and |log2 fold change| ≥ 1.0 (RNA-seq) or ≥0.5 (microarray). GSEA was performed using fgsea with MSigDB Hallmark gene sets. Pathway activity scores were calculated for predefined signatures, including TGF-β signaling, WNT/β-catenin, fibrosis, angiogenesis, and immune-related pathways. Statistical comparisons were performed using the Wilcoxon rank-sum test with Benjamini-Hochberg correction.

### ScRNA-seq analysis

Two scRNA-seq datasets (GSE123902 and GSE253013) were analyzed independently to investigate *AGTR1* expression patterns in the TME. Primary tumor samples from both datasets were processed using Seurat (v.4/v.5).^27^ Quality control filtering retained cells with 200–7,500 detected features and mitochondrial gene percentage <20%. Data were normalized using log-normalization, and 2,000 highly variable features were identified. Principal-component analysis (PCA) was performed, followed by graph-based clustering (Louvain algorithm, resolution 0.5) and UMAP visualization. Cell types were annotated using AddModuleScore with predefined marker signatures for tumor cells, T cells, B cells, macrophages, dendritic cells, and CAFs. *AGTR1*-positive cells were defined as those with detectable expression (>0). Differential expression analysis between *AGTR1*+ and *AGTR1*-populations within each cell type, particularly CAFs, was performed using the Wilcoxon rank-sum test. In CAFs, pathway enrichment analysis was conducted using fgsea with MSigDB Hallmark gene sets. CAF-specific signatures were evaluated and GO enrichment analysis was performed for CAF-specific DEGs using cluster profiler (BP, MF, CC ontologies, adjusted *p* value <0.05).^28^^,^^29^

### Clinical samples and patient data

Patients with advanced or recurrent NSCLC who received ICB monotherapy or combination immunotherapy (chemo-immunotherapy) as first-line treatment at Kansai Medical University Hospital between initial enrollment and March 2024 were included in this retrospective analysis. Patients with *EGFR* mutations or *ALK* rearrangements were excluded. The final cohort comprised 89 patients treated with ICB monotherapy, and 257 patients treated with chemo-immunotherapy. The data cutoff was October 2024, and the study period is 1 year. Baseline clinical characteristics collected included age, sex, ECOG performance status, clinical stage, metastatic sites, histological subtype, PD-L1 TPS, and concurrent use of ARBs. Progression-free survival (PFS) was calculated from the date of treatment initiation to the date of disease progression or to the last follow-up, whichever occurred first. For IHC analysis, FFPE tissue samples obtained before treatment initiation were available from 16 patients who received combination immunotherapy. These samples were used to assess *AGTR1* expression and TME characteristics. This study was conducted in accordance with the Helsinki Declaration of 1964 and its later versions, as well as the ethical guidelines for clinical studies. The institutional review board at our institution approved this study (approval no. 2021306).

### Immunohistochemistry staining

FFPE tumor tissues underwent IHC staining for *AGTR1* and phospho-SMAD2/3. IHC staining was performed using the BOND-III automated immunostainer (Leica Biosystems). The primary antibodies used were as follows: anti-*AGTR1* polyclonal antibody (1:200 dilution, catalog 25343-1-AP, Proteintech) and anti-phospho-SMAD2/SMAD3 (Ser465/467/423/425) polyclonal antibody (1:200 dilution, catalog PA5-110155, Thermo Fisher Scientific). Staining was visualized using standard detection systems. The expression levels were quantified using the H-score method, calculated as the sum of the percentage of cells at each staining intensity level multiplied by the intensity score (0 = negative, 1 = weak, 2 = moderate, and 3 = strong), resulting in a range of 0–300. H-scores were evaluated by at least two investigators for both tumor and stromal compartments. *AGTR1* expression and phospho-SMAD2/3 staining were assessed to determine receptor expression patterns and TGF-β pathway activation status, respectively.

### Statistical analysis

All statistical analyses were performed using R software and GraphPad Prism (v.10.4.1). Continuous variables were compared between the two groups using the Mann-Whitney *U* test. Categorical variables were assessed using Fisher’s exact test. PFS was estimated using the Kaplan-Meier method, and the log rank test was used to evaluate differences between groups. Univariate and multivariate Cox proportional hazards regression analyses were performed to identify prognostic factors. HRs with 95% CIs were calculated. Forest plots were generated to visualize the effect sizes and CIs of variables included in the Cox regression models. All statistical tests were two-sided, and *p* values <0.05 were considered statistically significant.

## Data and code availability

He publicly available datasets analyzed in this study can be freely downloaded from the GEO database (accession numbers: GSE32863, GSE253013, and GSE123902) and TCGA database (TCGA-LUAD and TCGA-LUSC). The datasets generated or analyzed during the current study are not publicly available; however, they are available from the corresponding author upon reasonable request.

## Acknowledgments

We would like to thank the study participants and their families. This research did not receive any specific grants from public, commercial, or not-for-profit funding agencies. This study was conducted in accordance with the Helsinki Declaration of 1964 and later versions and with the Ethical Guidelines for Clinical Studies. This study was approved by the institutional review board of 10.13039/501100012412Kansai Medical University (approval no. 2021306). Claude (Anthropic) was used for language editing and assistance with manuscript preparation.

## Author contributions

T.I. contributed to conceptualization, methodology, formal analysis, investigation, data curation, original draft preparation, review and editing of the manuscript, and visualization; T.K. contributed to conceptualization, methodology, investigation, review and editing of the manuscript, visualization, supervision, and project administration; K.A., M.K., N.M., Y.N., K.F., Y.Okuno, K.K., Y.Okazaki, K.Nakanishi, Y.S., K.Y., K.Nakahama, Y.T., U.K., Y.Y., S.I., H.Y., and T.S. contributed to data curation, investigation, and review and editing of the manuscript. All authors take full responsibility for all content.

## Declaration of interests

T.K. reports having received research support from multiple pharmaceutical companies, including MSD, AstraZeneca, Amgen, Boehringer Ingelheim, Daiichi Sankyo, Takeda Pharmaceutical, and Bristol Myers Squibb. He has also been compensated by AstraZeneca, Ono Pharmaceutical, MSD, Nippon Kayaku, Takeda Pharmaceutical, Eli Lilly, Bristol Myers Squibb, Chugai Pharmaceutical, and Pfizer for delivering lectures. H.Y. has obtained lecture-related honoraria from Boehringer Ingelheim, Chugai Pharmaceutical, Nippon Kayaku, Taiho Pharmaceutical, Eli Lilly, Takeda Pharmaceutical, and Bristol Myers Squibb. S.I. reports receiving lecture or speaking fees from AstraZeneca, Bristol Myers Squibb, Ono Pharmaceutical, Taiho Pharmaceutical, Chugai Pharmaceutical, Boehringer Ingelheim, Eli Lilly, Takeda Pharmaceutical, Pfizer, MSD, Daiichi Sankyo, Amgen, and Novartis. AstraZeneca and Chugai Pharmaceutical have awarded him research funding, and he has served in consulting or advisory roles for AstraZeneca, Chugai Pharmaceutical, and Daiichi Sankyo. All other co-authors state that they have no relevant conflicts of interest.

## Supplemental information

Document S1. Figure S1

Document S2. Article plus supplemental information
