# AGTR1 signaling contributes to tumor immunity and therapy response in non-small cell lung cancer

**DOI:** 10.1016/j.omton.2026.201336

**Published:** 2026-09-10

**Authors:** Tatsuki Ikoma, Keigo Araki, Mai Kitagawa, Natsuno Makihara, Yutaro Nagata, Kazuki Fujii, Yukiko Okuno, Keisuke Kamisako Yuta Okazaki, Kentaro Nakanishi, Yume Sanada, Kiyori Yoshida, Kahori Nakahama, Yuki Takeyasu, Utae Katsushima, Yuta Yamanaka, Satoshi Ikeda, Hiroshige Yoshioka, Toshio Shimizu, Takayasu Kurata

## Main text

(Molecular Therapy: Oncology *34*, 1–11; September 2026)

In the originally published version of this article, Figures 1C and 1D displayed the same heatmap. During revision of the figures in response to peer review, the heatmap intended for Figure 1C was inadvertently replaced by a duplicate of the heatmap shown in Figure 1D; Figure 1D is correct as published. To correct this error, Figure 1 has been replaced so that Figure 1C now displays the intended heatmap, which is consistent with the methods, results text, and figure legend as originally published. This error does not affect the results or conclusions of the article. The authors apologize for this error and any inconvenience it may have caused..

**Figure:**
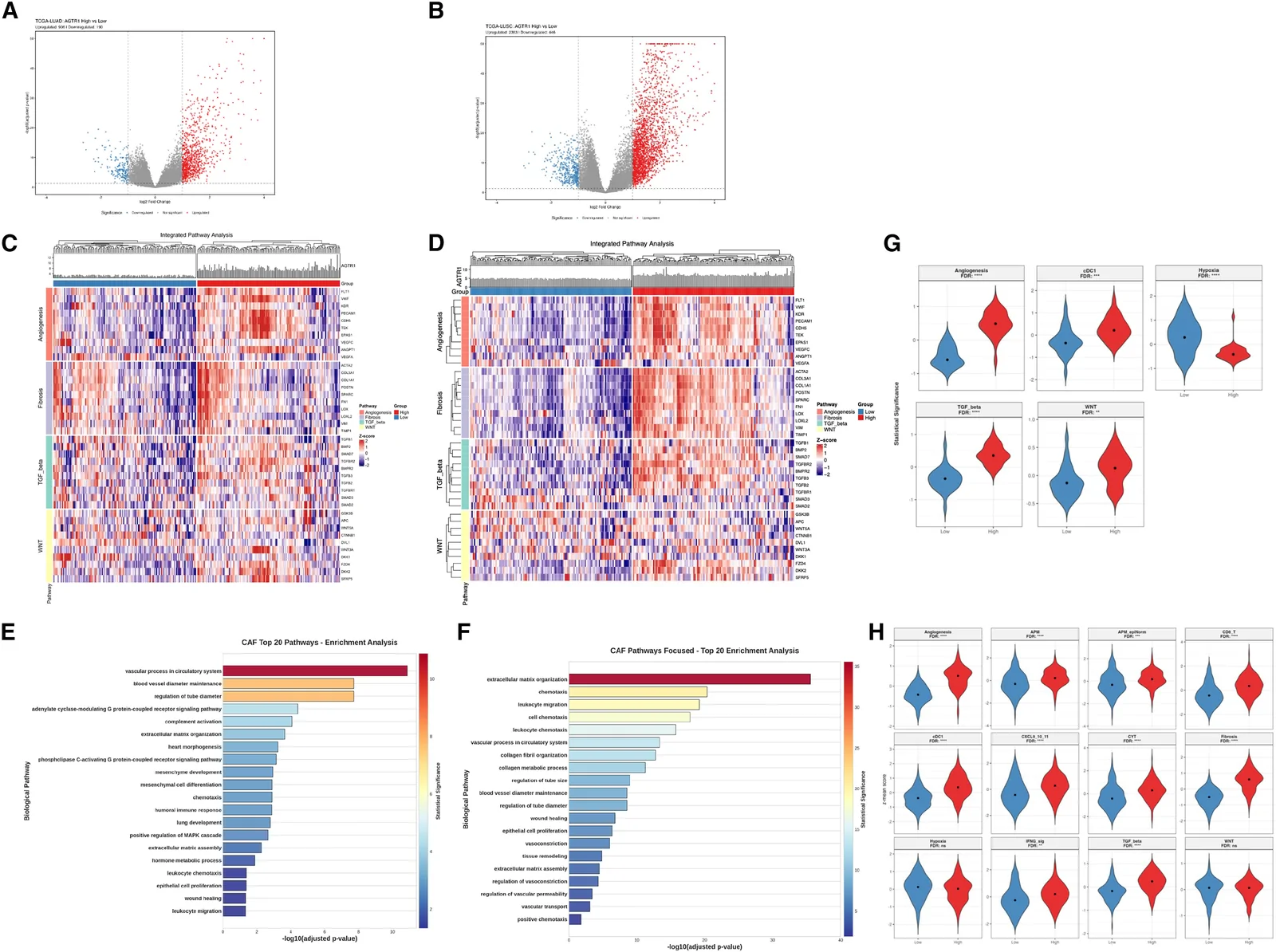
Figure 1. *AGTR1* expression analysis and pathway enrichment in lung cancer datasets (corrected)

**Figure:**
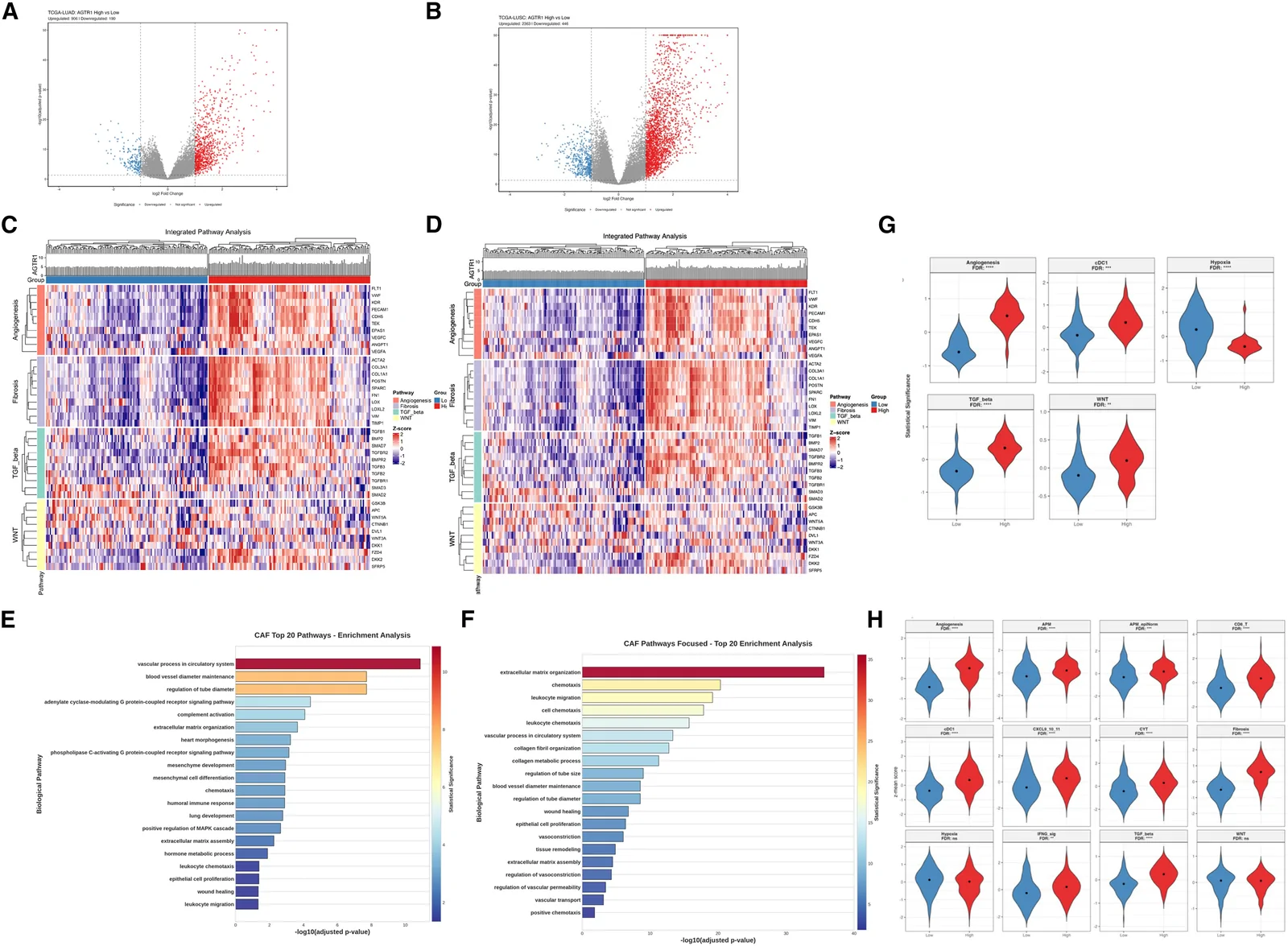
Figure 1. *AGTR1* expression analysis and pathway enrichment in lung cancer datasets (original)

