# Supplementary material for: AGTR1 signaling contributes to tumor immunity and therapy response in non-small cell lung cancer
Source: Mol Ther Oncol. 2026 May 29;34(3):201253. doi: 10.1016/j.omton.2026.201253 (PMC13293692; doi:10.1016/j.omton.2026.201253)
Supplement: Document S1. Figure S1 [file mmc1.pdf]

OMTON, Volume *34*

Supplemental information

AGTR1 signaling contributes to tumor immunity and therapy response in non-small cell lung cancer

Tatsuki Ikoma, Keigo Araki, Mai Kitagawa, Natsuno Makihara, Yutaro Nagata, Kazuki Fujii, Yukiko Okuno, Keisuke Kamisako, Yuta Okazaki, Kentaro Nakanishi, Yume Sanada, Kiyori Yoshida, Kahori Nakahama, Yuki Takeyasu, Utae Katsushima, Yuta Yamanaka, Satoshi Ikeda, Hiroshige Yoshioka, Toshio Shimizu, and Takayasu Kurata

Figure S1

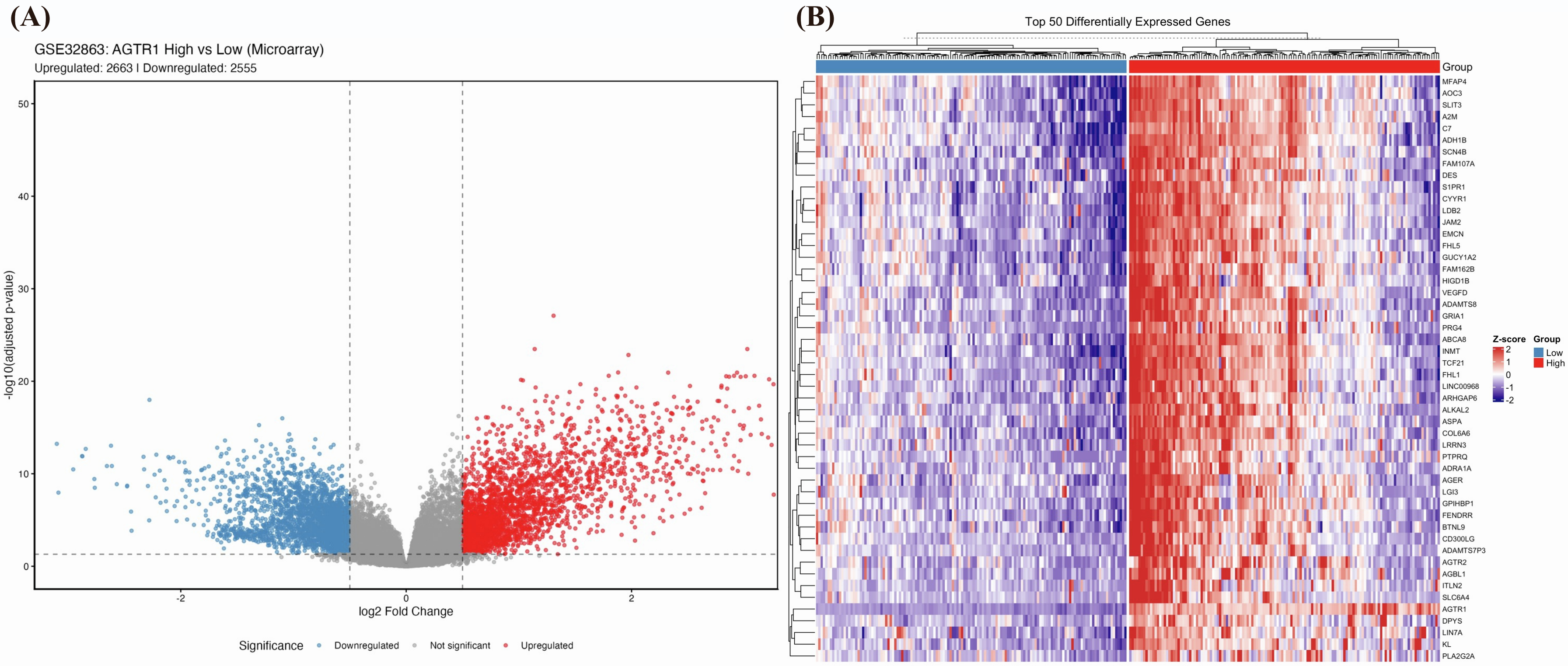
(A)
GSE32863: AGTR1 High vs Low (Microarray)
Upregulated: 2663 | Downregulated: 2555
-log10(adjusted p-value)
log2 Fold Change
Significance
Downregulated
Not significant
Upregulated
(B)
Top 50 Differentially Expressed Genes
Group
MFAP4
AOC3
SLIT3
A2M
C7
ADH1B
SCN4B
FAM107A
DES
S1PR1
CYYR1
LDB2
JAM2
EMCN
FHL5
GUCY1A2
FAM162B
HIGD1B
VEGFD
ADAMTS8
GRIA1
PRG4
ABCA8
INMT
TCF21
FHL1
LINC00968
ARHGAP6
ALKAL2
ASPA
COL6A6
LRRN3
PTPRQ
ADRA1A
AGER
LGI3
GPIHBP1
FENDRR
BTNL9
CD300LG
ADAMTS7P3
AGTR2
AGBL1
ITLN2
SLC6A4
AGTR1
DPYS
LIN7A
KL
PLA2G2A
Z-score
Low
High

(C)

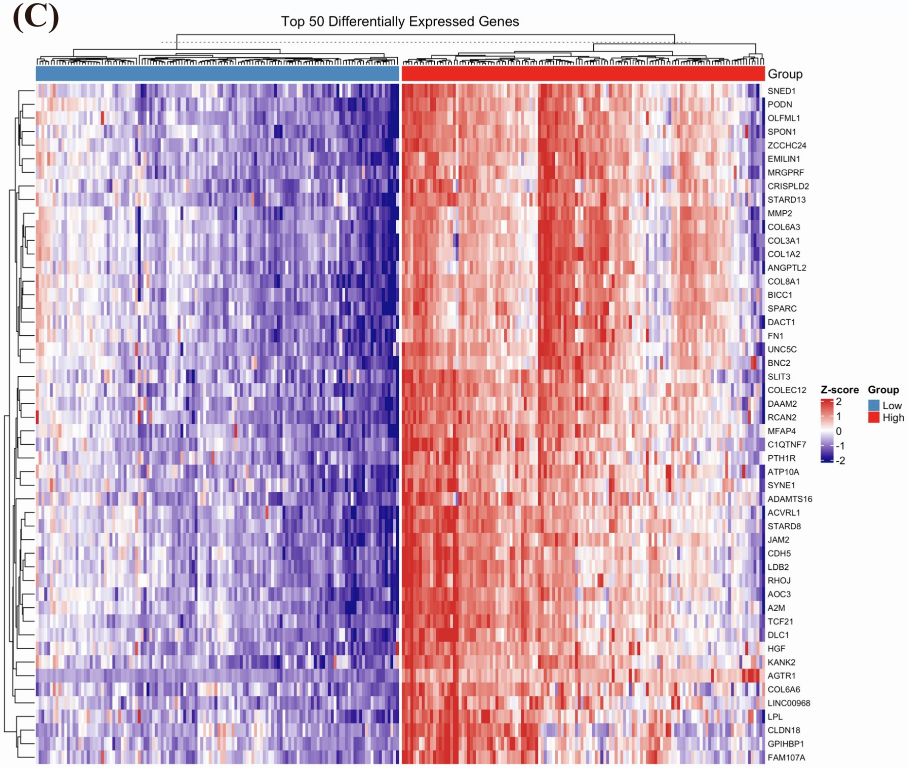
Top 50 Differentially Expressed Genes
Group
SNED1
PODN
OLFML1
SPON1
ZCCHC24
EMILIN1
MRGPRF
CRISPLD2
STARD13
MMP2
COL6A3
COL3A1
COL1A2
ANGPTL2
COL8A1
BICC1
SPARC
DACT1
FN1
UNC5C
BNC2
SLIT3
COLEC12
DAAM2
RCAN2
MFAP4
C1QTNF7
PTH1R
ATP10A
SYNE1
ADAMTS16
ACVRL1
STARD8
JAM2
CDH5
LDB2
RHOJ
AOC3
A2M
TCF21
DLC1
HGF
KANK2
AGTR1
COL6A6
LINC00968
LPL
CLDN18
GPIHBP1
FAM107A
Z-score
2
1
0
-1
-2
Group
Low
High

(D)

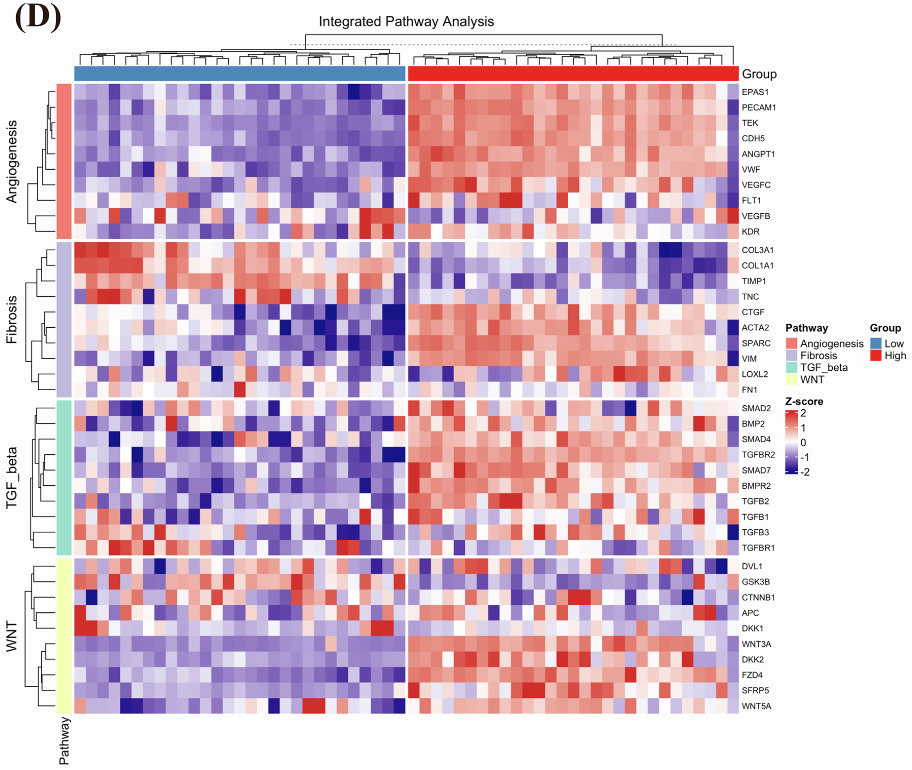
Integrated Pathway Analysis
Group
Angiogenesis
Fibrosis
TGF_beta
WNT
Pathway
EPAS1
PECAM1
TEK
CDH5
ANGPT1
VWF
VEGFC
FLT1
VEGFB
KDR
COL3A1
COL1A1
TIMP1
TNC
CTGF
ACTA2
SPARC
VIM
LOXL2
FN1
SMAD2
BMP2
SMAD4
TGFBR2
SMAD7
BMPR2
TGFB2
TGFB1
TGFB3
TGFBR1
DVL1
GSK3B
CTNNB1
APC
DKK1
WNT3A
DKK2
FZD4
SFRP5
WNT5A
Pathway
Angiogenesis
Fibrosis
TGF_beta
WNT
Group
Low
High
Z-score
2
1
0
-1
-2

(E)

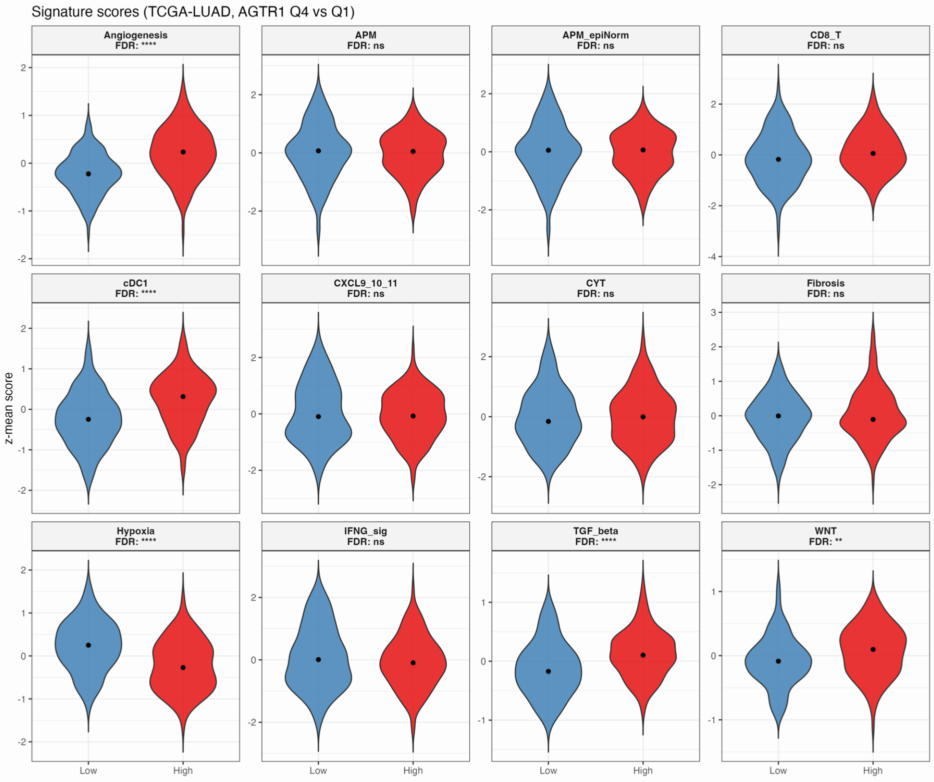
Signature scores (TCGA-LUAD, AGTR1 Q4 vs Q1)
Angiogenesis
FDR: ****
APM
FDR: ns
APM_epiNorm
FDR: ns
CD8_T
FDR: ns
cDC1
FDR: ****
CXCL9_10_11
FDR: ns
CYT
FDR: ns
Fibrosis
FDR: ns
Hypoxia
FDR: ****
IFNG_sig
FDR: ns
TGF_beta
FDR: ****
WNT
FDR: **
z-mean score
Low
High

(F)

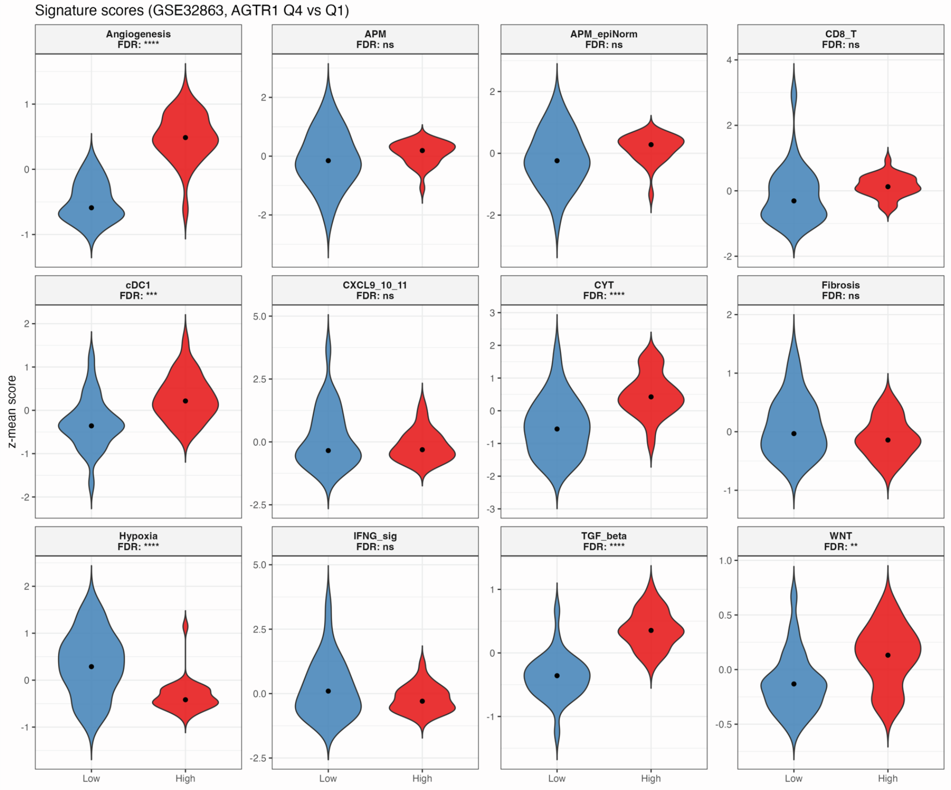
Signature scores (GSE32863, AGTR1 Q4 vs Q1)
Angiogenesis
FDR: ****
APM
FDR: ns
APM_epiNorm
FDR: ns
CD8_T
FDR: ns
cDC1
FDR: ***
CXCL9_10_11
FDR: ns
CYT
FDR: ****
Fibrosis
FDR: ns
Hypoxia
FDR: ****
IFNG_sig
FDR: ns
TGF_beta
FDR: ****
WNT
FDR: **
z-mean score
Low
High

(G)

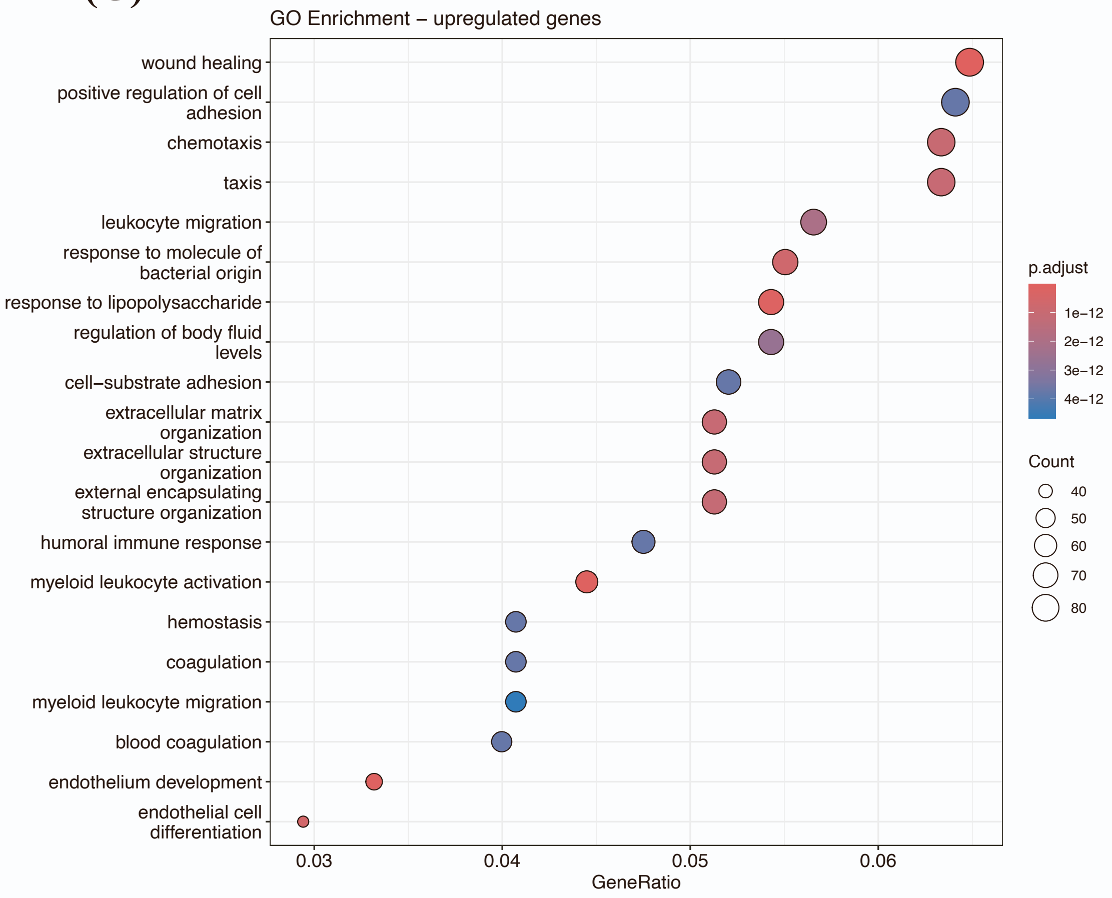

GO Enrichment – upregulated genes
wound healing
positive regulation of cell adhesion
chemotaxis
taxis
leukocyte migration
response to molecule of bacterial origin
response to lipopolysaccharide
regulation of body fluid levels
cell–substrate adhesion
extracellular matrix organization
extracellular structure organization
external encapsulating structure organization
humoral immune response
myeloid leukocyte activation
hemostasis
coagulation
myeloid leukocyte migration
blood coagulation
endothelium development
endothelial cell differentiation
0.03
0.04
0.05
0.06
GeneRatio
p.adjust
1e−12
2e−12
3e−12
4e−12
Count
40
50
60
70
80

Figure S1.
Additional validation analyses in the GSE32863 dataset and extended pathway assessments

Volcano plot showing differentially expressed genes between AGTR1-high and AGTR1-low groups in the GSE32863 dataset (Fig. A). Red dots indicate upregulated genes (n=2,663), blue dots indicate downregulated genes (n=2,555), and gray dots indicate non-significant genes. Vertical dashed lines represent log2 fold change thresholds, and the horizontal dashed line represents the adjusted p-value threshold. Heatmap showing the top 50 differentially expressed genes in the GSE32863 dataset (Fig. B). Samples are grouped by AGTR1 expression levels (low vs. high). The color scale represents Z-scores of gene expression, with red indicating high expression and blue indicating low expression. The other heatmap shows the top 50 differentially expressed genes, with hierarchical clustering, from the GSE32863 dataset (Fig. C). Notable genes include collagens, matrix metalloproteinases (MMP2), and various extracellular matrix-related genes. In Fig. D, the heatmap shows integrated pathway analysis of the GSE32863 dataset. Samples are grouped by AGTR1 expression. Four major pathway categories are shown: Angiogenesis, Fibrosis, TGF-beta, and WNT. The color scale represents pathway activity scores. Violin plots comparing pathway signature scores between AGTR1-low (blue) and AGTR1-high (red) groups in the TCGA-LUAD dataset, in Fig. E. Multiple pathway signatures are displayed, including Angiogenesis, APM, APM_epithelium, CD8 T cells, cDC1, CXCL9_10_11, CYT, Fibrosis, Hypoxia, IFNG_sig, TGF-beta, and WNT pathways. Statistical significance is denoted for significant comparisons. In Fig. F, the violin plots comparing pathway signature scores between AGTR1-low (blue) and AGTR1-high (red) groups in the GSE32863 dataset. The identical pathway signatures in panel E are analyzed for validation purposes. Note the differences in IFNG_sig and WNT pathway distributions compared to TCGA-LUAD. Bubble plot showing GO enrichment analysis of upregulated genes in the GSE32863 dataset. The x-axis represents GeneRatio, and the y-axis lists enriched biological processes. Bubble size indicates gene count, and color gradient represents adjusted p-value (p.adjust), with red indicating higher significance. Top enriched pathways include wound healing, chemotaxis, cell adhesion, leukocyte migration, and extracellular matrix organization.
