# Supplementary material for: AGTR1 signaling contributes to tumor immunity and therapy response in non-small cell lung cancer
Source: Mol Ther Oncol. 2026 May 29;34(3):201253. doi: 10.1016/j.omton.2026.201253 (PMC13293692; doi:10.1016/j.omton.2026.201253)
Supplement: Document S2. Article plus supplemental information [file mmc2.pdf]

Molecular Therapy
Oncology
Original Article

AGTR1 signaling contributes to tumor immunity and therapy response in non-small cell lung cancer

Tatsuki Ikoma,[1,2,3] Keigo Araki,[1] Mai Kitagawa,[1] Natsuno Makihara,[1] Yutaro Nagata,[1] Kazuki Fujii,[1] Yukiko Okuno,[1] Keisuke Kamisako,[1] Yuta Okazaki,[1] Kentaro Nakanishi,[1] Yume Sanada,[1] Kiyori Yoshida,[1] Kahori Nakahama,[1] Yuki Takeyasu,[1] Utae Katsushima,[1] Yuta Yamanaka,[1,2] Satoshi Ikeda,[1] Hiroshige Yoshioka,[1,4] Toshio Shimizu,[2] and Takayasu Kurata[1]

[1]Department of Thoracic Oncology, Kansai Medical University, 2-5-1, Shinmachi, Hirakata-city, Osaka 573-1010, Japan; [2]Department of New Experimental Therapeutics, Kansai Medical University Hospital, 2-3-1, Shinmachi, Hirakata-city, Osaka 573-1191, Japan; [3]Department of Clinical Oncology, Kansai Medical University Hospital, 2-3-1, Shinmachi, Hirakata-city, Osaka 573-1191, Japan; [4]Department of Respiratory Medicine and Clinical Immunology, Dokkyo Medical University Saitama Medical Center, Saitama, Japan

Angiotensin II receptor type 1 (*AGTR1*) has emerged as a potential modulator of the tumor microenvironment, yet its role in the immunotherapy response in non-small cell lung cancer (NSCLC) remains unclear. We performed a comprehensive analysis using bulk RNA sequencing and single-cell RNA sequencing (scRNA-seq) datasets. Functional validation included IHC for *AGTR1* and phospho-SMAD2/3 co-localization. The clinical impact of angiotensin receptor blocker (ARB) use was assessed in patients receiving immune checkpoint blockade (ICB) alone or combined with chemo-immunotherapy. *AGTR1*-high expression in NSCLC was associated with pronounced pathway modulation, including significant upregulation of TGF-β, etc. scRNA-seq analysis revealed that *AGTR1* was predominantly localized in cancer-associated fibroblasts (CAFs). IHC validation in NSCLC specimens (n = 14) demonstrated a strong correlation between stromal AGTR1 and phospho-SMAD2/3 expression and ARB-treated patients showed significantly reduced stromal *AGTR1* and pSMAD2/3 expression. Clinically, ARB treatment showed no benefit in ICB monotherapy but significantly improved PFS in chemo-immunotherapy (HR = 0.70, p = 0.01), especially in non-squamous (non-Sq) histology with PD-L1≥1% (HR = 0.52, p = 0.01). AGTR1 is predominantly expressed in CAFs and is suggestive of a correlation with TGF-β-related immunosuppressive features in NSCLC. The combination of ARB with chemo-immunotherapy may enhance therapeutic efficacy through targeting that axis in CAFs, specifically in non-Sq NSCLC patients.

INTRODUCTION

Immune checkpoint blockade (ICB) possesses the ability to activate patients' endogenous T cells, thereby promoting tumor elimination.[1] These therapeutic agents, initially introduced for melanoma treatment, have now established themselves as a cornerstone of standard care across numerous cancer types.[1] Furthermore, the advent of ICBs has led to an increase in cases achieving long-term survival, undoubtedly establishing these agents as revolutionary therapeutics in cancer treatment. However, not all patients respond to these drugs. Unlike conventional therapies, ICBs represent a unique class of agents in that even within the same cancer type, there exists a dichotomy between responder and non-responder populations.

The tumor microenvironment (TME), which surrounds the cancer, has gained attention as a key determinant of this heterogeneity in treatment response among homogeneous cancer populations.[2] The TME includes complex components, such as immune cells, including T cells and diverse stromal cells including fibroblasts.[3] These stromal cells have been shown to suppress tumor immunity, leading to limited anticancer effects when treated with not only ICB monotherapy but also chemo-immunotherapy.[4,5] Fibroblasts, especially cancer-associated fibroblasts (CAFs), produce collagen fibers in the peritumoral region, promoting fibrosis progression and creating an environment that hinders immune cell infiltration. Therefore, overcoming fibrosis may represent a crucial step toward successful immunotherapy.

Non-small cell lung cancer (NSCLC) is one of the malignancies for which immunotherapy has become standard treatment.[6] As mentioned above, numerous cases have demonstrated dramatically improved prognosis following the advent of immunotherapy.

Received 8 January 2026; accepted 27 May 2026;
https://doi.org/10.1016/j.omton.2026.201253.

Correspondence: Tatsuki Ikoma, Department of Thoracic Oncology, Kansai Medical University, 2-5-1, Shinmachi, Hirakata-city, Osaka 573-1010, Japan.
E-mail: ikoma.tat@kmu.ac.jp
Correspondence: Takayasu Kurata, Department of Thoracic Oncology, Kansai Medical University, 2-5-1, Shinmachi, Hirakata-city, Osaka-fu 573-1191, Japan.
E-mail: kurata.tak@kmu.ac.jp

© 2026 The Author(s).
Published by Elsevier Inc. on behalf of The American Society of Gene and Cell Therapy.
This is an open access article under the CC BY license (http://creativecommons.org/licenses/by/4.0/).

However, there also exists a population that remains refractory to immunotheraphy with persistently poor prognosis. In NSCLC, CAFs have been reported to correlate with prognosis and treatment efficacy, making the targeting of CAFs an urgent challenge.[7] Angiotensin II receptor 1 (*AGTR1*) has been identified in multiple reports as a potential target for CAF regulation.[8–10] Angiotensin receptor blockers (ARBs) are agents that inhibit the *AGTR1* receptor. ARBs were initially developed as antihypertensive drugs and are widely prescribed medications. Recent studies have explored their potential for drug repositioning in cancer treatment beyond their use as antihypertensives.[10,11] However, most investigations remain limited to preclinical data, and studies examining the impact of ARBs on antitumor effects in first-line immunotherapy for NSCLC remain scarce. Therefore, we investigated the role of *AGTR1* in tumor and stromal cells, as well as its impact on treatment effectiveness in NSCLC, utilizing both public databases and institutional data.

RESULTS

AGTR1 expression demonstrates distinct pathway modulation in lung adenocarcinoma

Bulk RNA sequencing (RNA-seq) analysis revealed that, regardless of histological subtype (LUAD or lung squamous cell carcinoma, LUSC), *AGTR1*-high expression groups showed differential expression of diverse genes compared to low-expression groups (Figures 1A and 1B). A total of 1,096 and 2,809 DEGs were identified in LUAD and LUSC, respectively, of which 558 (16.7%) were shared between the two subtypes, with 95.7% showing concordant direction of change. In LUAD, the top upregulated DEGs besides *AGTR1* included *SLC6A4*, *INMT*, *ADAMTS7P3*, *GPIHBP1*, *TCF21*, *VEGFD*, *AGER*, *PRG4*, *ADH1B*, and *ABCA8*, whereas in LUSC, *SPON1*, *COLEC12*, *TCF21*, *DLC1*, *ZCCHC24*, *CRISPLD2*, *AOC3*, *JAM2*, *DAAM2*, and *SNED1* were among the top upregulated genes. Notably, *TCF21*, a known fibroblast marker, was shared between both subtypes. Heatmap analysis also revealed distinct patterns in gene expression between the two groups for genes associated with representative pathways, including angiogenesis, fibrosis, WNT/β-catenin signaling, and transforming growth factor β (TGF-β) signaling (Figures 1C and 1D).

Furthermore, gene set enrichment analysis (GSEA) analysis revealed that pathways involved in angiogenesis, extracellular matrix (ECM), and fibrosis were significantly enriched in the *AGTR1*-high expression group relative to the *AGTR1*-low group (Figures 1E and 1F). We performed pathway signature analyses comparing *AGTR1*-high versus *AGTR1*-low expression groups across three cohorts (The Cancer Genome Atlas [TCGA]-LUAD, TCGA-LUSC, and an independent adenocarcinoma cohort GSE32863) (Figures 1G and 1H; Figure S1F). The LUAD dataset demonstrated the most pronounced pathway modulation, with *AGTR1*-high tumors showing significant upregulation of angiogenesis (diff = 0.45, adjusted $p = 3.36 \times 10^{-12}$), TGF-β (diff = 0.25, adjusted $p = 9.14 \times 10^{-6}$), hypoxia, and the conventional dendritic cell 1 (cDC1) signature, all of which exhibited distinct bimodal distributions with minimal overlap between groups. The independent adenocarcinoma cohort corroborated these findings, with similar upregulation of angiogenesis, TGF-β, cytolytic activity, and cDC1, though it notably showed a decreased hypoxia signature.

In contrast, the LUSC dataset exhibited a distinct pattern of pathway activation. While a broader range of pathways reached statistical significance, including APM (diff = 0.49, adjusted $p = 9.53 \times 10^{-6}$), CD8+ T cells (diff = 0.74, adjusted $p = 4.46 \times 10^{-11}$), CXCL9/10/11 (diff = 0.52, adjusted $p = 9.44 \times 10^{-6}$), cytolytic activity, and fibrosis (diff = 1.11, adjusted $p = 2.64 \times 10^{-36}$), these immune-related signatures were not significantly associated with *AGTR1* expression in the LUAD dataset. These findings suggest that *AGTR1* expression in LUAD is predominantly linked to TGF-β-dependent stromal remodeling, whereas in LUSC, *AGTR1* correlates with a more diverse microenvironmental profile encompassing both stromal and immune compartments. Across all cohorts, angiogenesis and TGF-β signatures were consistently upregulated, and cDC1 levels were elevated in all cohorts.

AGTR1 is predominantly expressed in CAFs and regulates TME remodeling

To determine the cellular localization of *AGTR1* in the lung adenocarcinoma microenvironment, we analyzed two independent single-cell RNA sequencing (scRNA-seq) datasets. Uniform manifold approximation and projection (UMAP) visualization demonstrated that *AGTR1* expression was predominantly localized to CAFs, with minimal expression detected in a small subset of tumor cells (Figure 2A).

To elucidate the functional role of *AGTR1* in CAFs, we compared pathway enrichment between *AGTR1*-positive and *AGTR1*-negative CAF populations. Bar plot analysis revealed that *AGTR1*+ CAFs exhibited significant enrichment of multiple pathways involved in microenvironment remodeling (Figure 2B). Especially, scRNA-seq analysis revealed that the TGF-β signaling pathway was robustly upregulated in *AGTR1*+ CAFs (Figure 2C). *AGTR1*+ CAFs exhibited significantly elevated TGF-β activated signature scores (mean difference = 0.026, effect size = 0.116, adjusted p value = 0.037). However, statistical significance was not achieved across all pathways. In contrast, a comparative analysis of *AGTR1*+ versus *AGTR1*-tumor cells across both datasets revealed no consistent pattern of pathway enrichment, suggesting that the stromal compartment primarily drives *AGTR1*-mediated effects on the microenvironment.

IHC analysis of *AGTR1* and TGF-β pathway activation

Our bioinformatics analysis of publicly available datasets suggested an association between *AGTR1* expression and activity of the TGF-β signaling pathway. To further explore this association in clinical specimens, we performed immunohistochemical (IHC) analysis using pathological specimens from a subset of non-squamous NSCLC patients treated at our institution (n = 14; ARB-treated, n = 5; non-ARB-treated, n = 9).

We evaluated *AGTR1* and phosphorylated SMAD2/3 (pSMAD2/3), a marker of TGF-β pathway activation, by IHC (Figure 3A). *AGTR1*

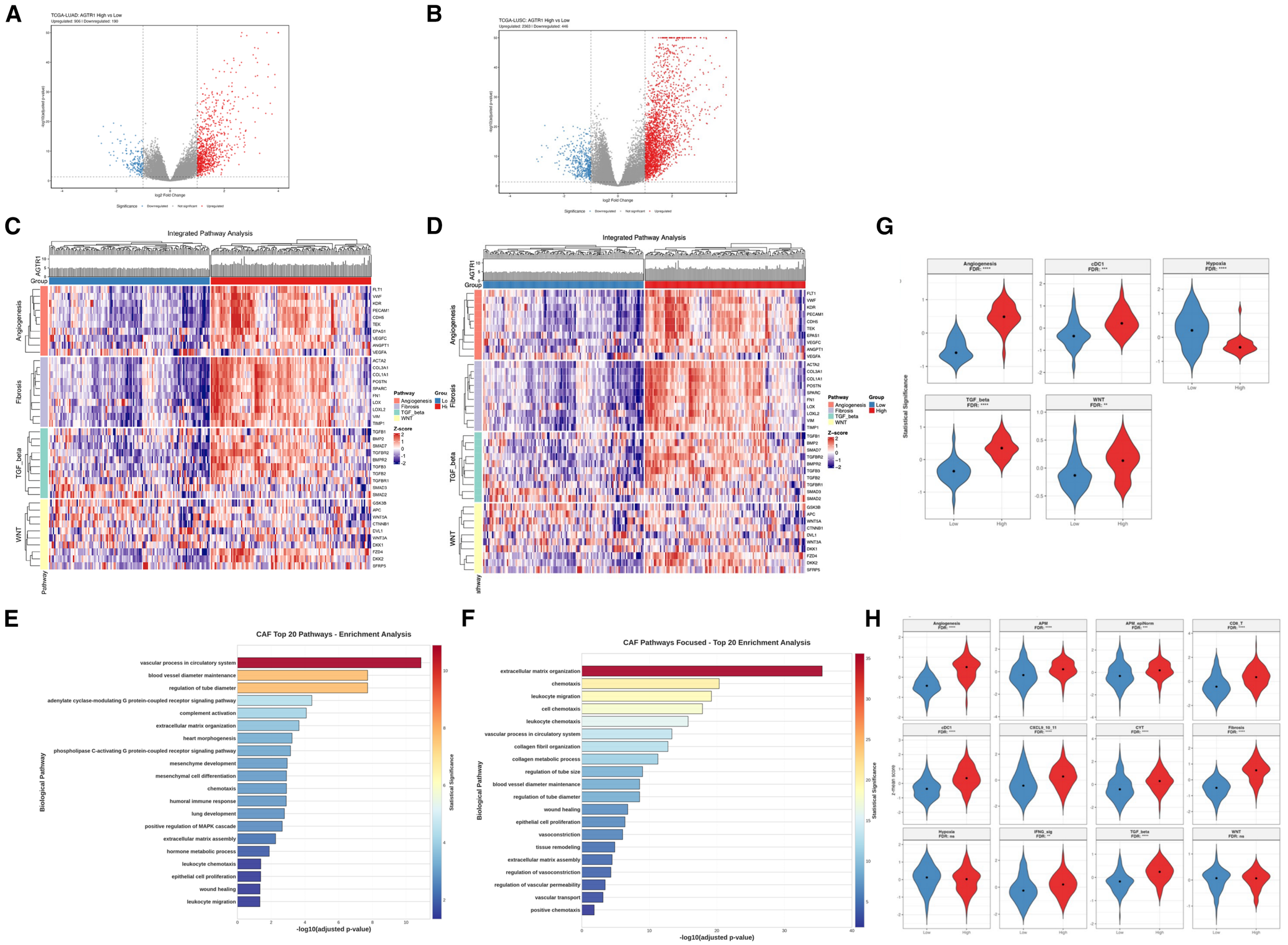

Figure 1. *AGTR1* expression analysis and pathway enrichment in lung cancer datasets

(A and B) Volcano plots showing differentially expressed genes between *AGTR1*-high and *AGTR1*-low groups in TCGA-LUAD (A) and TCGA-LUSC (B). Red dots indicate upregulated genes, blue dots indicate downregulated genes, and gray dots indicate non-significant genes. Vertical dashed lines represent log2 fold change thresholds (±1), and the horizontal dashed line represents the adjusted p value threshold (0.05). (C and D) Heatmaps displaying integrated pathway, including angiogenesis, fibrosis, TGF-beta, and WNT/b-catenin, analysis of differentially expressed genes in LUAD (C) and LUSC (D). Samples are grouped by AGTR1 expression levels (low vs. high). The color scale represents pathway activity scores (Z-scores), with red indicating high activity and blue indicating low activity. (E and F) Bar plots showing Gene Ontology enrichment analysis of biological pathways in LUAD (E) and LUSC (F). The x axis represents—log10 (adjusted p value), and the y axis lists enriched biological processes. The color gradient indicates statistical significance, with red indicating higher significance. Key enriched pathways include vascular processes, extracellular matrix organization, and chemotaxis. (G and H) Violin plots comparing pathway signature scores between *AGTR1*-low (blue) and *AGTR1*-high (red) groups in LUAD (G) and LUSC (H). Multiple pathway signatures are displayed, including Angiogenesis, conventional dendritic cell 1 (cDC1), Hypoxia, TGF-beta, and WNT/b-catenin pathways. Black dots represent median values, and error bars indicate interquartile ranges. Statistical significance is denoted as: *$p < 0.05$, **$p < 0.01$, ***$p < 0.001$, ****$p < 0.0001$. FDR, false discovery rate.

and pSMAD2/3 were stained on separate slides prepared from the same formalin-fixed paraffin-embedded (FFPE) tumor blocks of each patient; multiplex co-staining was not performed in this study. The staining intensity was assessed using a four-tier scoring system as illustrated in the figures. Based on our previous analyses indicating predominant *AGTR1* expression in CAFs, we focused our evaluation on the tumor stromal compartment.

In the stromal regions, we observed a significant positive correlation between *AGTR1* expression and pSMAD2/3 expression (Spearman's $\rho = 0.86$, $p = 7.22 \times 10^{-5}$; Figure 3B). Representative histological images are shown in Figure 3C. Notably, patients who received ARB treatment exhibited significantly lower stromal *AGTR1* expression than those without ARB treatment (H-score: 86.0 ± 5.4 vs. 155.6 ± 26.5, p value <0.001; Figure 3D). Furthermore, pSMAD2/3 expression was also considerably reduced in ARB-treated patients (H-score: 89.0 ± 18.8 vs. 158.9 ± 41.9, p value <0.01; Figure 3E), consistent with a correlative association between stromal *AGTR1* expression and TGF-β pathway activity in the TME.

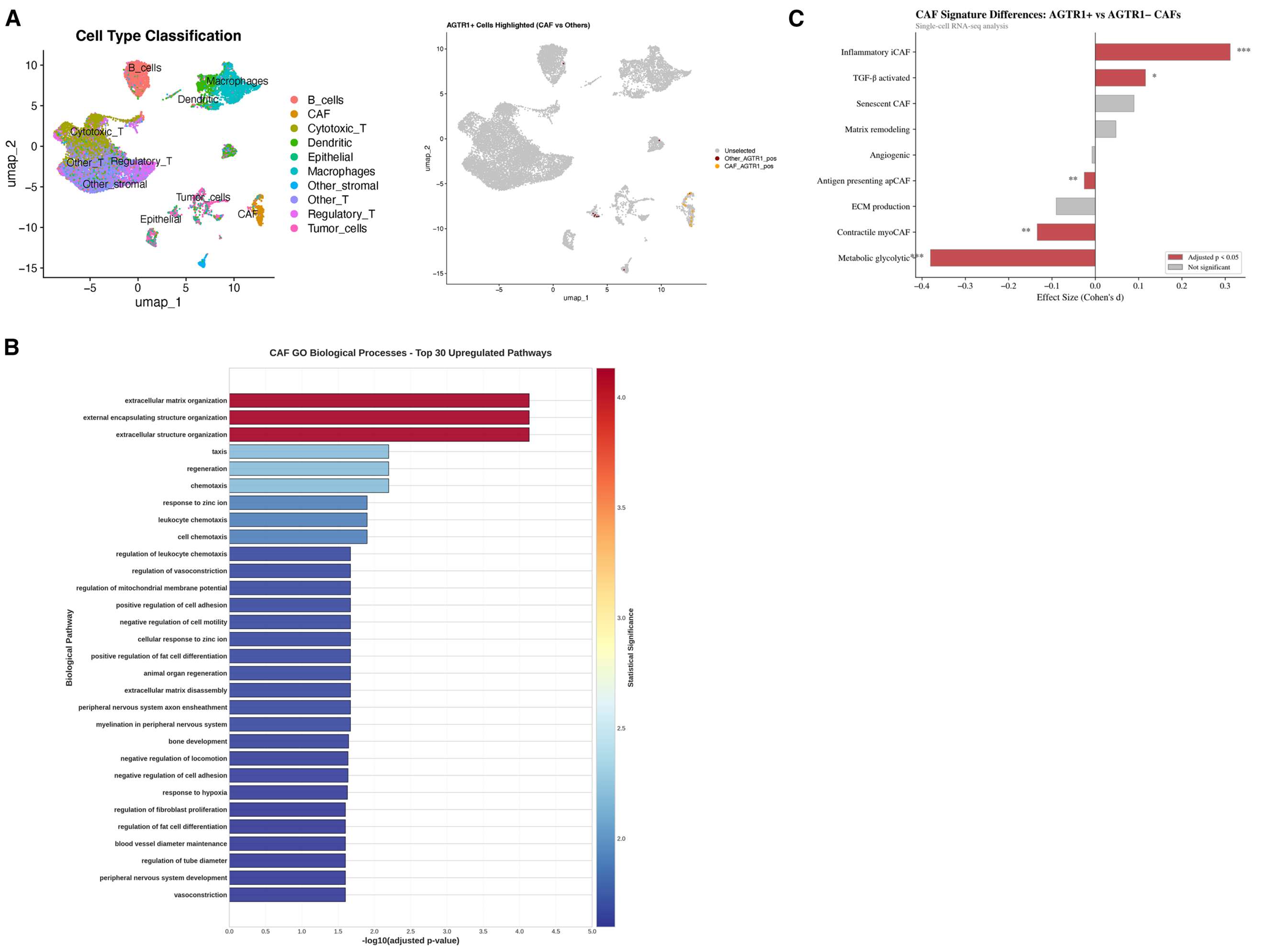

Figure 2. Single-cell RNA sequencing analysis reveals *AGTR1* expression predominantly in cancer-associated fibroblasts

(A) Left: UMAP plot showing cell type classification in lung cancer tissue. Eleven distinct cell populations are identified, including B cells, CAF, cytotoxic T cells, dendritic cells, epithelial cells, macrophages, other stromal cells, other T cells, regulatory T cells, and tumor cells. Right: UMAP plot highlighting *AGTR1*-positive cells, with orange dots representing CAF cells expressing *AGTR1* and red dots representing other cell types expressing *AGTR1*. Gray dots indicate *AGTR1*-negative cells. (B) Horizontal bar plot showing GO enrichment analysis of biological pathways in the single-cell dataset. The *x* axis represents −log10(adjusted *p* value), and the *y* axis lists enriched biological processes. The color gradient indicates statistical significance, with red indicating the highest importance. Top enriched pathways include extracellular matrix organization, external encapsulating structure organization, and various chemotaxis-related processes. (C) Bar plot showing effect sizes (Cohen's d) of CAF-related signature scores comparing *AGTR1*-positive and *AGTR1*-negative CAFs from single-cell RNA-seq analysis.

Clinical outcomes and impact of ARB treatment on the effectiveness of immunotherapy

We evaluated the impact of ARB treatment on clinical outcomes using data from patients treated at our institution. First, we assessed whether ARB administration influenced PD-L1 tumor proportion score (TPS) expression. No significant difference in PD-L1 TPS was observed between ARB-treated and non-treated patients (33.7% ± 37.2% vs. 37.0% ± 39.1%, p value = 0.83), indicating that ARB does not directly modulate tumor PD-L1 expression (Figure 4A).

Patient baseline characteristics for the ICB monotherapy (n = 89) and chemo-immunotherapy (n = 207) cohorts are summarized in Table 1. The median observation period for this clinical research cohort is 12.5 months (0.19–98.9 months). Within each treatment modality, there were no significant imbalances in baseline characteristics between patients with and without ARB treatment. In the ICB monotherapy group, ARBs included telmisartan (n = 7), olmesartan (n = 6), valsartan (n = 3), and others (n = 9). In the chemo-immunotherapy group, the most frequently used agents were candesartan (n = 16), telmisartan (n = 15), azilsartan (n = 10), and others (n = 37). In the ICB monotherapy cohort (n = 89; ARB n = 25, non-ARB n = 64), ARB treatment did not confer a substantial benefit in progression-free survival (PFS) (median PFS: 4.0 vs. 4.1 month, hazard ratio (HR, 1.06), 95% confidence interval (CI, 0.64–1.76,

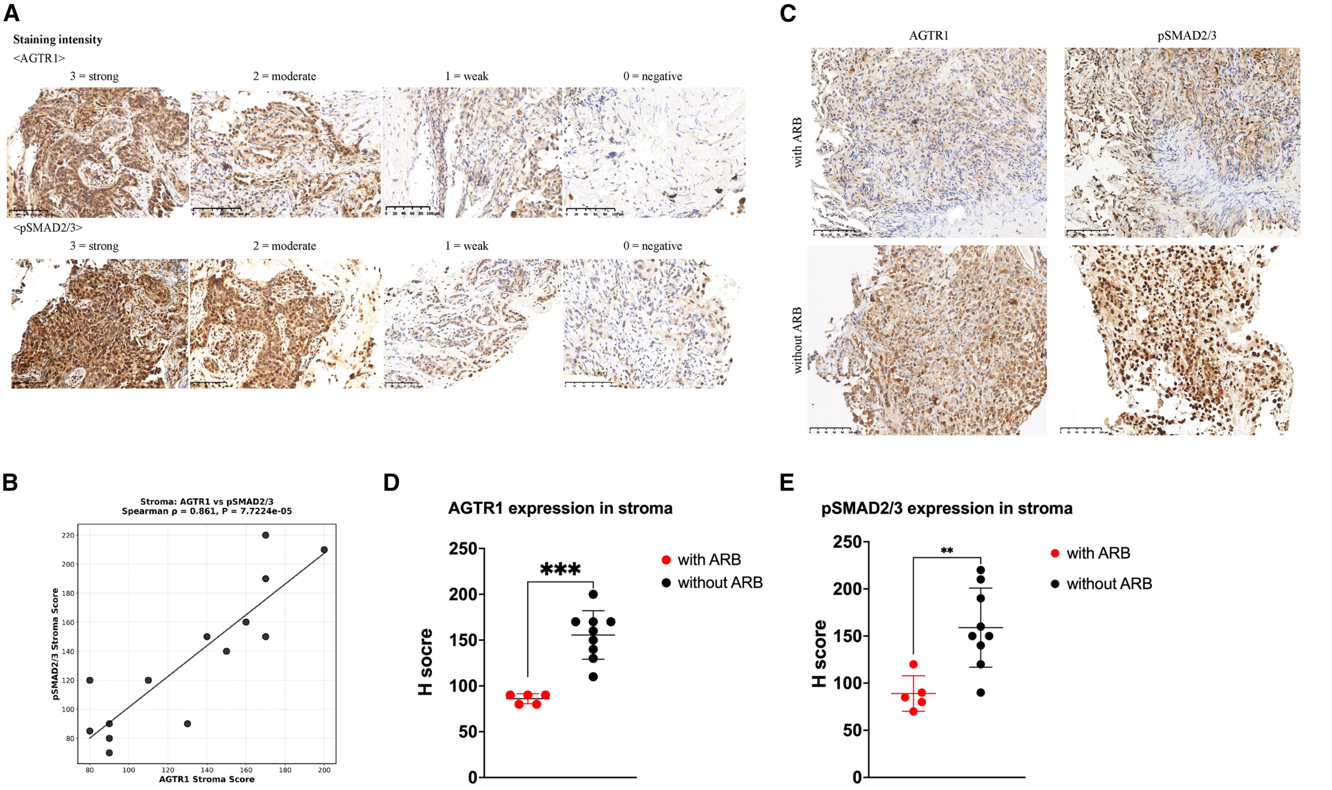

Figure 3. Immunohistochemical analysis of *AGTR1* and pSMAD2/3 expression in lung cancer tissues and correlation with ARB treatment

(A) Representative immunohistochemical images showing *AGTR1* and pSMAD2/3 staining intensity in lung cancer stromal cells. Staining intensity is scored as: 3 = strong, 2 = moderate, 1 = weak, 0 = negative. Scale bars, 100 μm. (B) The scatterplot shows a positive correlation between *AGTR1* and pSMAD2/3 expression in stromal cells ($n = 14$ patients). The Spearman's correlation coefficient is $\rho = 0.861$, and the p value is 7.7224e−05. Each dot represents an individual patient sample, and the line shows the linear regression fit. (C) Representative immunohistochemical images comparing *AGTR1* (left) and pSMAD2/3 (right) expression in stromal cells between patients with ARB treatment (top) and without ARB treatment (bottom). (D) Scatterplot showing *AGTR1* expression levels (H-score) in stromal cells. Red dots represent patients with ARB treatment ($n = 5$), and black dots represent patients without ARB treatment ($n = 9$). Horizontal lines indicate mean ± standard deviation. *AGTR1* expression is significantly lower in the ARB group compared to the non-ARB group (***$p < 0.001$, Mann-Whitney *U* test). (E) Scatterplot showing pSMAD2/3 expression levels (H-score) in stromal cells. Red dots represent patients with ARB treatment ($n = 5$), and black dots represent patients without ARB treatment ($n = 9$). Horizontal lines indicate mean ± standard deviation. pSMAD2/3 expression is significantly lower in the ARB group compared to the non-ARB group (**$p < 0.01$, Mann-Whitney *U* test).

p value = 0.80; Figure 4B). Subgroup analysis using a forest plot revealed no patient populations that clearly benefited from ARB in this setting (Figure 4D).

In contrast, patients receiving chemo-immunotherapy ($n = 257$; ARB $n = 78$, non-ARB $n = 179$) with concurrent ARB treatment demonstrated significantly prolonged PFS compared to those without ARB (median PFS: 8.1 vs. 5.8 months, HR 0.70, 95% CI 0.53–0.93, p value = 0.01; Figure 4C). Subgroup analysis identified specific populations that derived greater benefit from ARB, particularly patients with PD-L1 TPS ≥1% and non-squamous histology (Figure 4E). In the same population, ultivariate Cox regression analysis, adjusting for age, sex, Eastern Cooperative Oncology Group performance status (ECOG PS), PD-L1 TPS, and presence of liver metastasis, confirmed that ARB treatment independently predicted improved PFS in the chemo-immunotherapy cohort (HR 0.52, 95% CI 0.31–0.89, $p = 0.01$; Table 2). When the analysis was restricted to patients with non-squamous histology and PD-L1 TPS ≥1%, the PFS benefit associated with ARB was further prolonged, with median PFS of 16.3 months in the ARB group compared to 5.8 months in the non-ARB group (HR 0.52, 95% CI 0.34–0.89, p value = 0.01; Figure 4F). In a complementary analysis, the objective response rate (ORR) was compared between ARB-treated and non-ARB-treated patients. In the ICB monotherapy group, ORR was similar between ARB-treated and non-ARB-treated patients (32.0% vs. 32.8%, $p > 0.99$). In the chemo-immunotherapy group, a numerically higher ORR was observed in ARB-treated patients (62.8% vs. 51.9%), although this difference did not reach statistical significance ($p = 0.13$), likely reflecting the limited sample size of the cohort.

DISCUSSION

We performed comprehensive bioinformatic analyses and clinical validation to elucidate the role of *AGTR1* in NSCLC and its therapeutic implications for immunotherapy. Our findings demonstrate that

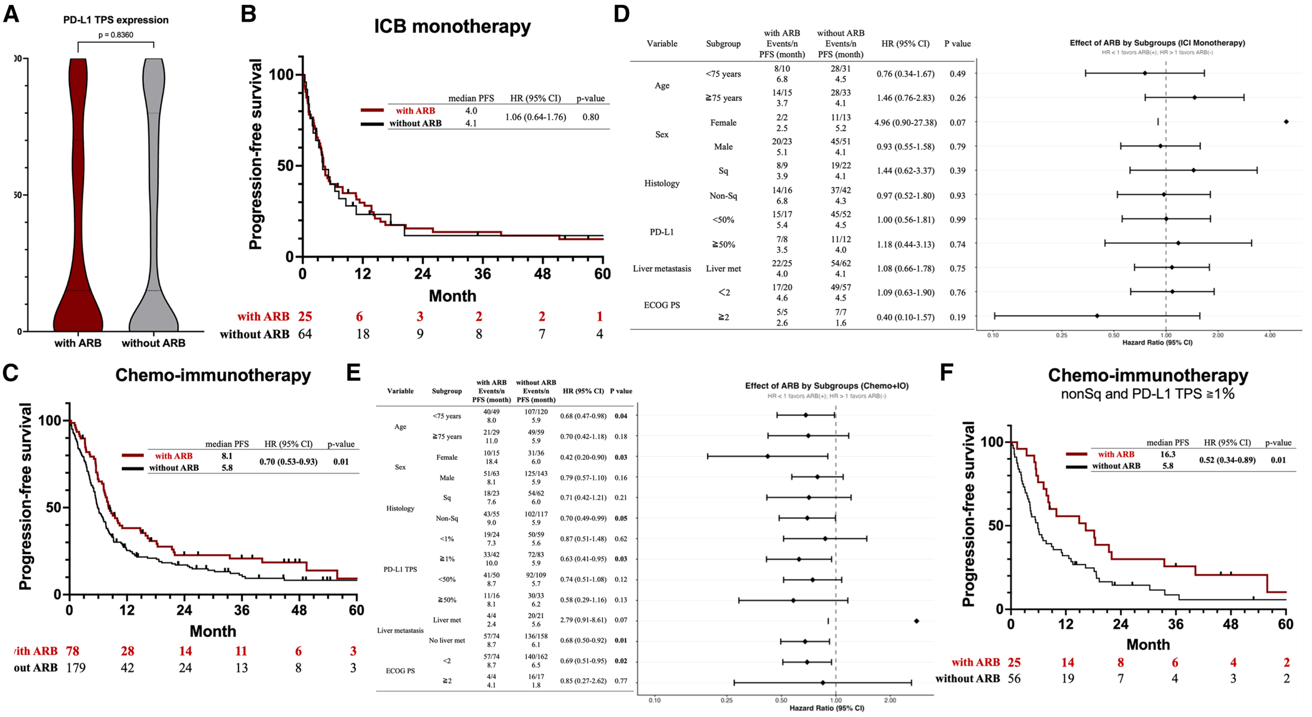

Figure 4. Clinical outcomes and progression-free survival analysis in lung cancer patients stratified by ARB treatment

(A) Violin plot comparing PD-L1 TPS distribution between patients with ARB treatment (red, $n = 91$) and without ARB treatment (black, $n = 206$) in the entire study cohort. The width of each violin represents the density of data points at different values. Black dots indicate median values, and error bars represent interquartile ranges. No significant difference was observed between the two groups (33.7 ± 37.2% vs. 37.0 ± 39.1%, $p = 0.83$, Mann-Whitney U test). (B) Kaplan-Meier curve showing PFS in patients treated with ICB monotherapy. The red line represents patients with ARB treatment ($n = 25$), and the black line represents patients without ARB treatment ($n = 64$). Median PFS: 4.0 months (with ARB) vs. 4.1 month (without ARB); HR = 1.06 (95% CI 0.64–1.76), $p = 0.80$ (log rank test). (C) Kaplan-Meier curve showing PFS in patients treated with chemo-immunotherapy. The red line represents patients with ARB treatment ($n = 78$), and the black line represents patients without ARB treatment ($n = 179$). Median PFS: 8.1 months (with ARB) vs. 5.8 months (without ARB); HR = 0.70 (95% CI 0.53–0.93), $p = 0.01$ (log rank test). (D) Forest plot showing subgroup analysis of ARB effect on PFS in patients receiving ICB monotherapy. Variables analyzed include age, sex, histology, PD-L1 expression, liver metastasis, and ECOG PS. Hazard ratios with 95% confidence intervals are displayed. No subgroup showed a significant benefit from ARB treatment. (E) Forest plot showing subgroup analysis of ARB effect on PFS in patients receiving chemo-immunotherapy. Variables analyzed include age, sex, histology, PD-L1 TPS, liver metastasis, and ECOG PS. Several subgroups showed significant benefit from ARB treatment, particularly in non-Sq histology ($p = 0.05$), PD-L1 TPS ≥1% ($p = 0.03$). (F) Kaplan-Meier curve showing PFS in chemo-immunotherapy patients with non-squamous histology and PD-L1 TPS ≥1%. The red line represents patients with ARB treatment ($n = 25$), and the black line represents patients without ARB treatment ($n = 56$). Median PFS: 16.3 months (with ARB) vs. 5.8 months (without ARB); HR = 0.52 (95% CI 0.34–0.89), $p = 0.01$.

AGTR1 is predominantly expressed in CAFs and is associated with stromal remodeling in the context of TGF-β signaling activation (Figure 5). Critically, ARB treatment significantly enhanced the effectiveness of chemo-immunotherapy in patients with non-squamous NSCLC and PD-L1 TPS ≥1%, whereas ICB monotherapy provided no benefit.

CAFs and immunotherapy resistance

CAFs represent critical determinants of immunotherapy resistance.[2] Based on the present findings, *AGTR1* may constitute a promising molecular target for overcoming CAF-mediated resistance mechanisms to immunotherapy. scRNA-seq and IHC demonstrated that *AGTR1* expression is predominantly localized to the tumor stroma, particularly in CAFs, rather than tumor cells. Of note, the proportion of *AGTR1*-positive CAFs appeared lower in scRNA-seq than in IHC, likely reflecting the known dropout of moderately expressed genes in scRNA-seq and the differences in sensitivity between mRNA and protein detection methods. CAFs not only establish physical barriers that restrict T cell infiltration but also activate multiple signaling pathways that attenuate the efficacy of immunotherapy.[12] However, consistent with our data, CAFs exhibit substantial heterogeneity in their functional properties.[13] We identified distinct molecular signatures between *AGTR1*-positive and *AGTR1*-negative CAF subpopulations, particularly in ECM-related pathway activation, indicating that CAFs, analogous to tumor cells, comprise heterogeneous populations with diverse functional states.[14] This heterogeneity presents significant challenges for pharmacological targeting of all CAF populations. Nevertheless, the identification of specific molecular markers, such as *AGTR1*-expressing CAFs, that contribute adversely to antitumor immunity represents a critical advancement toward the development of targeted therapeutic interventions.

Table 1. Baseline patient characteristics stratified by ARB treatment in ICB monotherapy and chemo-immunotherapy cohorts

	ICB monotherapy				Chemo-immunotherapy			
Variable	all *n* = 89 (%)	with ARB *n* = 25 (%)	without ARB *n* = 64 (%)	*p* value	all *n* = 257 (%)	with ARB *n* = 78 (%)	without ARB *n* = 179 (%)	*p* value
Age	–	–	–		–	–	–	
<75	41(46)	10 (40)	31 (48)	0.49	169 (66)	49 (63)	120 (67)	0.56
≧75	48 (54)	15 (60)	33 (52)		88 (34)	29 (37)	59 (33)	
Sex	–	–	–		–	–	–	
Male	74 (83)	23 (92)	51 (80)	0.21	206 (80)	63 (81)	143 (80)	0.99
Female	15 (17)	2 (8)	13 (20)		51 (20)	15 (19)	36 (20)	
ECOG PS	–	–	–		–	–	–	
<2	77 (87)	20 (80)	57 (89)	0.30	236 (92)	74 (95)	162 (91)	0.32
≧2	12 (13)	5 (20)	7 (11)		21 (8)	4 (5)	17 (9)	
Tissue	–	–	–		–	–	–	
Sq	31 (35)	9 (36)	22 (34)	0.99	85 (33)	23 (29)	62 (35)	0.47
Non-Sq	58 (65)	16 (64)	42 (66)		172 (67)	55 (71)	117 (65)	
Stage	–	–	–		–	–	–	
III/IV	63 (71)	17 (68)	46 (72)	0.79	187 (73)	56 (72)	131 (73)	0.87
Recurrence	26 (29)	8 (32)	18 (28)		70 (27)	22 (28)	48 (27)	
PD-L1 TPS	–	–	–		–	–	–	
≧50%	69 (78)	17 (68)	52 (81)		49 (19)	16 (21)	33 (18)	
1∼49%	20 (22)	8 (32)	12 (19)	0.25	76 (30)	26 (33)	50 (28)	0.76
<1%	–	–	–		83 (32)	24 (31)	59 (33)	
Unknown	–	–	–		49 (19)	12 (15)	37 (21)	
Metastasis	–	–	–		–	–	–	
Liver	2 (2)	0	2 (3)	–	25 (10)	4 (5)	21 (12)	–
Bone	27 (30)	8 (32)	19 (30)		82 (32)	25 (32)	57 (32)	
Brain	8 (9)	2 (8)	6 (9)		36 (14)	11 (14)	25 (14)	
CTLA-4	–	–	–		–	–	–	
Yes	–	–	–	–	16 (6)	4 (5)	12 (7)	0.78
No	–	–	–		241 (94)	74 (95)	167 (93)	
Bevacizumab	–	–	–		–	–	–	
Yes	–	–	–	–	13 (5)	4 (5)	9 (5)	0.99
No	–	–	–		244 (95)	74 (95)	170 (95)	

ARB, angiotensin receptor blocker; ECOG PS, Eastern Cooperative Oncology Group performance status; ICB, immune checkpoint blockade; Sq, Squamous cell carcinoma; PD-L1 TPS, PD-L1 tumor proportion score.

AGTR1 expression, TGF-β pathway activity, and therapeutic implications

The inhibitory effect of ARBs on TGF-β signaling is well-established in non-oncological settings, most notably in Marfan syndrome, where losartan attenuates TGF-β-driven aortic remodeling.[15] In the oncology context, several clinical trials have explored the combination of ARBs with conventional cancer therapies; losartan has been investigated as a stromal-modifying agent in combination with FOLFIRINOX and chemoradiation in locally advanced pancreatic cancer, demonstrating improved R0 resection rates.[16] Furthermore, retrospective studies have suggested that ARB or ACEI use is associated with improved outcomes in several cancer types, including colorectal and renal cell carcinoma.[17] However, the role of ARBs in combination with ICB remains largely unexplored. Our results demonstrate that *AGTR1* expression correlated with TGF-β-related transcriptional and IHC features in the TME. Previous investigations have reported that *AGTR1* signaling can promote fibrotic changes and an immunologically “cold” phenotype, findings that are consistent with our observations.[10,18] The novelty of the present study lies in demonstrating that *AGTR1* expression in NSCLC is predominantly localized to CAFs, that *AGTR1*+ CAFs showed a correlative association with TGF-β pathway activity, and that concomitant ARB use during chemo-immunotherapy correlates

Table 2. Univariate and multivariate analysis of progression-free survival in patients with non-squamous histology and PD-L1 TPS ≥1% receiving chemo-immunotherapy

	Chemo-immunotherapy			
	Univariate		Multivariate	
Variable	HR (95% CI)	*p* value	HR (95% CI)	*p* value
Age				
≧75 (ref, <75)	1.06 (0.65-1.75)	0.80	–	–
Sex				
Male (ref, female)	1.63 (0.86-3.08)	0.13	–	–
ECOG PS				
≧2 (ref, <2)	2.06 (0.97-4.37)	0.05	–	–
PD-L1 TPS				
≧50% (ref, < 50%)	1.16 (0.71-1.87)	0.54	–	–
Liver metastasis				
No (ref, Yes)	0.62 (0.28-1.36)	0.23	–	–
ARB				
Yes (ref, No)	0.52 (0.31-0.89)	**0.01**	0.52 (0.31-0.89)	**0.01**

HR, hazard ratio; CI, confidence interval.

with improved PFS in non-squamous NSCLC patients with PD-L1 TPS ≥1%. These findings provide a TME-specific hypothesis for repurposing ARBs as a candidate stromal-targeting strategy in combination with immunotherapy, warranting further functional and prospective clinical validation. The lower *AGTR1* immunostaining observed in tumors from patients receiving ARB treatment (Figure 3) may reflect long-term disruption of a positive feedback loop in which angiotensin II signaling through *AGTR1* sustains its own transcription. Chronic ARB-mediated receptor blockade may attenuate this autocrine signaling, potentially leading to gradual transcriptional downregulation of *AGTR1* in CAFs. Alternatively, the direct binding of ARBs to *AGTR1* may partially interfere with antibody epitope accessibility, resulting in reduced IHC detection rather than a true decrease in protein expression. These interpretations remain speculative and require functional validation in future studies.

Differential response patterns in ICB monotherapy and histological subtypes

Analysis of public datasets and clinical cohorts identified two distinct patient subpopulations in which the additive effect of *AGTR1*-targeting ARBs was limited: patients receiving ICB monotherapy and those with squamous cell carcinoma (Sq) patients undergoing chemo-immunotherapy. Regarding ICB monotherapy, our clinical cohort showed no significant improvement in PFS with or without ARB, and subgroup analyses did not reveal any apparent benefit. These findings suggest that *AGTR1* inhibition has been less effective in ICB monotherapy settings. Several mechanisms may explain this observation. First, while ARBs may suppress fibrosis progression, their capacity to reverse established fibrotic architecture may appear limited.[18] Second, *AGTR1* inhibition may paradoxically activate hypoxia-related pathways. Our data revealed enhanced hypoxia signatures in *AGTR1*-low expression groups. Hypoxia pathway activation promotes immune evasion and the development of cold TMEs, potentially attenuating ICB efficacy.[19,20] Third, *AGTR1* inhibition alone is insufficient to induce antigen presentation pathways. Our analysis revealed unchanged or decreased cDC1 and APM-related signatures in *AGTR1*-low groups.[21] While *AGTR1* inhibition may create a more permissive

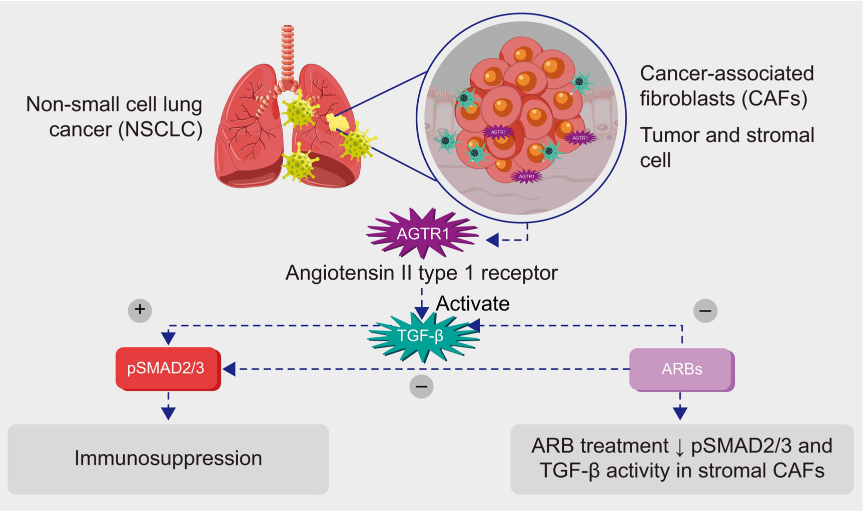

Figure 5. Schematic diagram depicting the regulatory role of *AGTR1* in the TME immune landscape of NSCLC

AGTR1 is predominantly expressed in CAFs, and *AGTR1* expression is suggestive of a correlation with TGF-β-related immunosuppressive features. ARBs, which predominantly inhibit *AGTR1*, may enhance immunotherapy efficacy by modulating CAFs, warranting further therapeutic development in this context.

environment for T cell recruitment, insufficient antigen presentation limits therapeutic improvements. Conversely, the addition of chemotherapy may enhance antigen presentation through immunogenic cell death, enabling fuller exploitation of the benefits of *AGTR1* inhibition.[22]

Histology-specific mechanisms in squamous cell carcinoma

In Sq patients receiving chemo-immunotherapy, therapeutic benefits remained limited. While angiogenesis- and TGF-β-related pathways were significantly downregulated in *AGTR1*-low Sq tumors, the magnitude of these changes might appear insufficient to translate into clear clinical benefits, particularly when contrasted with LUAD cohorts. Moreover, unlike LUAD, the WNT/β-catenin pathway remained activated in Sq cancers regardless of *AGTR1* expression status, potentially hindering enhancement of T cell-mediated antitumor activity.[23] These findings collectively suggest that *AGTR1* may serve as a novel therapeutic target to improve the efficacy of chemo-immunotherapy in non-Sq NSCLC.

Study limitations

This study has several limitations. First, functional analyses of *AGTR1* were primarily based on bioinformatic approaches, and experimental validation—including *AGTR1* knockdown or pharmacologic inhibition in CAFs followed by assessment of pSMAD2/3 and canonical TGF-β target genes—will be required in future studies to formally establish a functional *AGTR1*-TGF-β relationship. Second, the precise molecular mechanisms underlying *AGTR1*-related CAF activation remain to be fully elucidated. Third, this was a single-center retrospective study with limited sample size, requiring prospective clinical trials to confirm therapeutic impact. Fourth, although IHC and transcriptomic analyses consistently indicated an association between *AGTR1* expression and TGF-β signaling activation in CAFs, the present data remain correlative; multiplex co-staining was not performed in this study. Future investigations using multiplex immunofluorescence or spatial transcriptomics to directly demonstrate co-localization of *AGTR1* and pSMAD2/3 at single-cell resolution would be needed to substantiate a spatial relationship between *AGTR1* expression and TGF-β pathway activation.

Conclusion

This study demonstrates that *AGTR1* represents a poor prognostic factor in lung adenocarcinoma, is predominantly expressed in CAFs, and plays crucial roles in TME remodeling. Therapeutic strategies targeting *AGTR1* signaling may represent novel treatment options for lung adenocarcinoma, particularly in combination with chemo-immunotherapy for non-squamous histology.

MATERIALS AND METHODS

Human dataset acquisition

RNA-seq results were obtained, and analyses were conducted using both bulk RNA-seq datasets and scRNA-seq data. Regarding bulk RNA-seq, three datasets were acquired in total: the TCGA lung adenocarcinoma (TCGA-LUAD) and lung squamous cell carcinoma (TCGA-LUSC) datasets, along with the GSE32863 dataset.[24] For scRNA-seq, two datasets were obtained and analyzed: GSE123902[25] and GSE253013.[26]

Bulk transcriptome analysis

Three independent transcriptome datasets were analyzed to investigate *AGTR1* expression and its associated molecular signatures in NSCLC: RNA-seq data from TCGA-LUAD and TCGA-LUSC obtained via TCGAbiolinks, and microarray data from GSE32863 obtained from the Gene Expression Omnibus (GEO) database. Samples were stratified by *AGTR1* expression into quartiles, with Q1 (lowest 25%) and Q4 (highest 25%) selected for comparative analysis. Differential expression analysis was performed using DESeq2 for RNA-seq data and limma for microarray data. DEGs were defined as adjusted p value <0.05 and |log2 fold change| $\geq$ 1.0 (RNA-seq) or $\geq$0.5 (microarray). GSEA was performed using fgsea with MSigDB Hallmark gene sets. Pathway activity scores were calculated for predefined signatures, including TGF-β signaling, WNT/β-catenin, fibrosis, angiogenesis, and immune-related pathways. Statistical comparisons were performed using the Wilcoxon rank-sum test with Benjamini-Hochberg correction.

ScRNA-seq analysis

Two scRNA-seq datasets (GSE123902 and GSE253013) were analyzed independently to investigate *AGTR1* expression patterns in the TME. Primary tumor samples from both datasets were processed using Seurat (v.4/v.5).[27] Quality control filtering retained cells with 200–7,500 detected features and mitochondrial gene percentage <20%. Data were normalized using log-normalization, and 2,000 highly variable features were identified. Principal-component analysis (PCA) was performed, followed by graph-based clustering (Louvain algorithm, resolution 0.5) and UMAP visualization. Cell types were annotated using AddModuleScore with predefined marker signatures for tumor cells, T cells, B cells, macrophages, dendritic cells, and CAFs. *AGTR1*-positive cells were defined as those with detectable expression (>0). Differential expression analysis between *AGTR1*+ and *AGTR1*- populations within each cell type, particularly CAFs, was performed using the Wilcoxon rank-sum test. In CAFs, pathway enrichment analysis was conducted using fgsea with MSigDB Hallmark gene sets. CAF-specific signatures were evaluated and GO enrichment analysis was performed for CAF-specific DEGs using cluster profiler (BP, MF, CC ontologies, adjusted p value <0.05).[28,29]

Clinical samples and patient data

Patients with advanced or recurrent NSCLC who received ICB monotherapy or combination immunotherapy (chemo-immunotherapy) as first-line treatment at Kansai Medical University Hospital between initial enrollment and March 2024 were included in this retrospective analysis. Patients with *EGFR* mutations or *ALK* rearrangements were excluded. The final cohort comprised 89 patients treated with ICB monotherapy, and 257 patients treated with chemo-immunotherapy. The data cutoff was October 2024, and the study period is 1 year. Baseline clinical characteristics collected included age, sex, ECOG performance status, clinical stage, metastatic sites, histological subtype, PD-L1 TPS, and concurrent use of

ARBs. Progression-free survival (PFS) was calculated from the date of treatment initiation to the date of disease progression or to the last follow-up, whichever occurred first. For IHC analysis, FFPE tissue samples obtained before treatment initiation were available from 16 patients who received combination immunotherapy. These samples were used to assess *AGTR1* expression and TME characteristics. This study was conducted in accordance with the Helsinki Declaration of 1964 and its later versions, as well as the ethical guidelines for clinical studies. The institutional review board at our institution approved this study (approval no. 2021306).

Immunohistochemistry staining

FFPE tumor tissues underwent IHC staining for *AGTR1* and phospho-SMAD2/3. IHC staining was performed using the BOND-III automated immunostainer (Leica Biosystems). The primary antibodies used were as follows: anti-*AGTR1* polyclonal antibody (1:200 dilution, catalog 25343-1-AP, Proteintech) and anti-phospho-SMAD2/SMAD3 (Ser465/467/423/425) polyclonal antibody (1:200 dilution, catalog PA5-110155, Thermo Fisher Scientific). Staining was visualized using standard detection systems. The expression levels were quantified using the H-score method, calculated as the sum of the percentage of cells at each staining intensity level multiplied by the intensity score (0 = negative, 1 = weak, 2 = moderate, and 3 = strong), resulting in a range of 0–300. H-scores were evaluated by at least two investigators for both tumor and stromal compartments. *AGTR1* expression and phospho-SMAD2/3 staining were assessed to determine receptor expression patterns and TGF-β pathway activation status, respectively.

Statistical analysis

All statistical analyses were performed using R software and GraphPad Prism (v.10.4.1). Continuous variables were compared between the two groups using the Mann-Whitney *U* test. Categorical variables were assessed using Fisher's exact test. PFS was estimated using the Kaplan-Meier method, and the log rank test was used to evaluate differences between groups. Univariate and multivariate Cox proportional hazards regression analyses were performed to identify prognostic factors. HRs with 95% CIs were calculated. Forest plots were generated to visualize the effect sizes and CIs of variables included in the Cox regression models. All statistical tests were two-sided, and p values <0.05 were considered statistically significant.

DATA AND CODE AVAILABILITY

He publicly available datasets analyzed in this study can be freely downloaded from the GEO database (accession numbers: GSE32863, GSE253013, and GSE123902) and TCGA database (TCGA-LUAD and TCGA-LUSC). The datasets generated or analyzed during the current study are not publicly available; however, they are available from the corresponding author upon reasonable request.

ACKNOWLEDGMENTS

We would like to thank the study participants and their families. This research did not receive any specific grants from public, commercial, or not-for-profit funding agencies. This study was conducted in accordance with the Helsinki Declaration of 1964 and later versions and with the Ethical Guidelines for Clinical Studies. This study was approved by the institutional review board of Kansai Medical University (approval no. 2021306). Claude (Anthropic) was used for language editing and assistance with manuscript preparation.

AUTHOR CONTRIBUTIONS

T.I. contributed to conceptualization, methodology, formal analysis, investigation, data curation, original draft preparation, review and editing of the manuscript, and visualization; T.K. contributed to conceptualization, methodology, investigation, review and editing of the manuscript, visualization, supervision, and project administration; K.A., M.K., N.M., Y.N., K.F., Y.Okuno, K.K., Y.Okazaki, K.Nakanishi, Y.S., K.Y., K.Nakahama, Y.T., U.K., Y.Y., S.I., H.Y., and T.S. contributed to data curation, investigation, and review and editing of the manuscript. All authors take full responsibility for all content.

DECLARATION OF INTERESTS

T.K. reports having received research support from multiple pharmaceutical companies, including MSD, AstraZeneca, Amgen, Boehringer Ingelheim, Daiichi Sankyo, Takeda Pharmaceutical, and Bristol Myers Squibb. He has also been compensated by AstraZeneca, Ono Pharmaceutical, MSD, Nippon Kayaku, Takeda Pharmaceutical, Eli Lilly, Bristol Myers Squibb, Chugai Pharmaceutical, and Pfizer for delivering lectures. H.Y. has obtained lecture-related honoraria from Boehringer Ingelheim, Chugai Pharmaceutical, Nippon Kayaku, Taiho Pharmaceutical, Eli Lilly, Takeda Pharmaceutical, and Bristol Myers Squibb. S.I. reports receiving lecture or speaking fees from AstraZeneca, Bristol Myers Squibb, Ono Pharmaceutical, Taiho Pharmaceutical, Chugai Pharmaceutical, Boehringer Ingelheim, Eli Lilly, Takeda Pharmaceutical, Pfizer, MSD, Daiichi Sankyo, Amgen, and Novartis. AstraZeneca and Chugai Pharmaceutical have awarded him research funding, and he has served in consulting or advisory roles for AstraZeneca, Chugai Pharmaceutical, and Daiichi Sankyo. All other co-authors state that they have no relevant conflicts of interest.

SUPPLEMENTAL INFORMATION

Supplemental information can be found online at https://doi.org/10.1016/j.omton.2026.201253.

REFERENCES

1. Topalian, S.L., Hodi, F.S., Brahmer, J.R., Gettinger, S.N., Smith, D.C., McDermott, D.F., Powderly, J.D., Carvajal, R.D., Sosman, J.A., Atkins, M.B., et al. (2012). Safety, activity, and immune correlates of anti-PD-1 antibody in cancer. N. Engl. J. Med. *366*, 2443–2454.

2. Pitt, J.M., Marabelle, A., Eggermont, A., Soria, J.C., Kroemer, G., and Zitvogel, L. (2016). Targeting the tumor microenvironment: removing obstruction to anticancer immune responses and immunotherapy. Ann. Oncol. *27*, 1482–1492.

3. Lavie, D., Ben-Shmuel, A., Erez, N., and Scherz-Shouval, R. (2022). Cancer-associated fibroblasts in the single-cell era. Nat. Cancer *3*, 793–807.

4. Kieffer, Y., Hocine, H.R., Gentric, G., Pelon, F., Bernard, C., Bourachot, B., Lameiras, S., Albergante, L., Bonneau, C., Guyard, A., et al. (2020). Single-cell analysis reveals fibroblast clusters linked to immunotherapy resistance in cancer. Cancer Discov. *10*, 1330–1351.

5. Yan, Y., Sun, D., Hu, J., Chen, Y., Sun, L., Yu, H., Xiong, Y., Huang, Z., Xia, H., Zhu, X., et al. (2025). Multi-omic profiling highlights factors associated with resistance to immuno-chemotherapy in non-small-cell lung cancer. Nat. Genet. *57*, 126–139.

6. Reck, M., Rodríguez-Abreu, D., Robinson, A.G., Hui, R., Csőszi, T., Fülöp, A., Gottfried, M., Peled, N., Tafreshi, A., Cuffe, S., et al. (2021). Five-year outcomes with pembrolizumab versus chemotherapy for metastatic non-small-cell lung cancer with PD-L1 tumor proportion score ≥50%. J. Clin. Oncol. *39*, 2339–2349.

7. Cords, L., Engler, S., Haberecker, M., Rüschoff, J.H., Moch, H., de Souza, N., and Bodenmiller, B. (2024). Cancer-associated fibroblast phenotypes are associated with patient outcome in non-small cell lung cancer. Cancer Cell *42*, 396–412.e5.

8. Nakamura, K., Yaguchi, T., Ohmura, G., Kobayashi, A., Kawamura, N., Iwata, T., Kiniwa, Y., Okuyama, R., and Kawakami, Y. (2018). Involvement of local renin-angiotensin system in immunosuppression of tumor microenvironment. Cancer Sci. *109*, 54–64.

9. Li, J.H., Wu, X., Ni, X., Li, Y.X., Xu, L., Hao, X.Y., Zhao, W., Zhu, X.X., and Yin, X.Y. (2023). Angiotensin receptor blockers retard the progression and fibrosis via

inhibiting the viability of AGTR1+ CAFs in intrahepatic cholangiocarcinoma. Clin. Transl. Med. *13*, e1213.

10. Mei, J., Chu, J., Yang, K., Luo, Z., Yang, J., Xu, J., Li, Q., Zhang, Y., Zhang, Q., Wan, M., et al. (2024). Angiotensin receptor blocker attacks armored and cold tumors and boosts immune checkpoint blockade. J. Immunother. Cancer *12*, e009327.
11. Carey, R.M., Moran, A.E., and Whelton, P.K. (2022). Treatment of hypertension: a review. JAMA *328*, 1849–1861.
12. Henriques, A., Salvany-Celades, M., Nieto, P., Palomo-Ponce, S., Sevillano, M., Hernando-Momblona, X., Middendorp-Guerra, E., Llanses Martinez, M., Haak, E.M., Nieto, J., et al. (2025). TGF-β builds a dual immune barrier in colorectal cancer by impairing T cell recruitment and instructing immunosuppressive SPP1+ macrophages. Nat. Genet. *57*, 3050–3065.
13. Melchionna, R., Trono, P., Di Carlo, A., Di Modugno, F., and Nisticò, P. (2023). Transcription factors in fibroblast plasticity and CAF heterogeneity. J. Exp. Clin. Cancer Res. *42*, 347.
14. Han, J., Kim, E.G., Kim, B.Y., and Soung, N.K. (2025). Cancer-associated fibroblasts arising from endothelial-to-mesenchymal transition: induction factors, functional roles, and transcriptomic evidence. Biology *14*, 1403.
15. Brooke, B.S., Habashi, J.P., Judge, D.P., Patel, N., Loeys, B., and Dietz, H.C., 3rd (2008). Angiotensin II blockade and aortic-root dilation in Marfan's syndrome. N. Engl. J. Med. *358*, 2787–2795.
16. Murphy, J.E., Wo, J.Y., Ryan, D.P., Clark, J.W., Jiang, W., Yeap, B.Y., Drapek, L.C., Ly, L., Baglini, C.V., Blaszkowsky, L.S., et al. (2019). Total neoadjuvant therapy with FOLFIRINOX in combination with losartan followed by chemoradiotherapy for locally advanced pancreatic cancer: a phase 2 clinical trial. JAMA Oncol. *5*, 1020–1027.
17. Mc Menamin, Ú.C., Murray, L.J., Cantwell, M.M., and Hughes, C.M. (2012). Angiotensin-converting enzyme inhibitors and angiotensin receptor blockers in cancer progression and survival: a systematic review. Cancer Causes Control. *23*, 221–230.
18. Nakamura, K., Okuyama, R., and Kawakami, Y. (2020). Renin-angiotensin system in the tumor microenvironment. Adv. Exp. Med. Biol. *1277*, 105–114.
19. Robles-Oteíza, C., Hastings, K., Choi, J., Sirois, I., Ravi, A., Expósito, F., de Miguel, F., Knight, J.R., López-Giráldez, F., Choi, H., et al. (2025). Hypoxia is linked to acquired resistance to immune checkpoint inhibitors in lung cancer. J. Exp. Med. *222*, e20231106.
20. Liu, L., Wu, D., Qian, Z., Jiang, Y., You, Y., Wang, Y., Zhang, F., Ning, X., Mei, J., Iqbal, J., et al. (2025). Empowering hypoxia to convert cold tumors into hot tumors for breast cancer immunotherapy. Cell Death Discov. *11*, 381.
21. Maier, B., Leader, A.M., Chen, S.T., Tung, N., Chang, C., LeBerichel, J., Chudnovskiy, A., Maskey, S., Walker, L., Finnigan, J.P., et al. (2020). A conserved dendritic-cell regulatory program limits antitumour immunity. Nature *580*, 257–262.
22. Zhang, H., Li, S., Liu, S., Liao, Y., Liu, H., Yang, M., and Chen, P. (2025). Therapeutic targeting of cell death-immune crosstalk in cancer to rewire the tumor immune microenvironment. Mol. Cancer *24*, 277.
23. Clara, J.A., Monge, C., Yang, Y., and Takebe, N. (2020). Targeting signalling pathways and the immune microenvironment of cancer stem cells - a clinical update. Nat. Rev. Clin. Oncol. *17*, 204–232.
24. Selamat, S.A., Chung, B.S., Girard, L., Zhang, W., Zhang, Y., Campan, M., Siegmund, K.D., Koss, M.N., Hagen, J.A., Lam, W.L., et al. (2012). Genome-scale analysis of DNA methylation in lung adenocarcinoma and integration with mRNA expression. Genome Res. *22*, 1197–1211.
25. Laughney, A.M., Hu, J., Campbell, N.R., Bakhoum, S.F., Setty, M., Lavallée, V.P., Xie, Y., Masilionis, I., Carr, A.J., Kottapalli, S., et al. (2020). Regenerative lineages and immune-mediated pruning in lung cancer metastasis. Nat. Med. *26*, 259–269.
26. Xiang, H., Pan, Y., Sze, M.A., Wlodarska, M., Li, L., van de Mark, K.A., Qamar, H., Moure, C.J., Linn, D.E., Hai, J., et al. (2024). Single-cell analysis identifies NOTCH3-mediated interactions between stromal cells that promote microenvironment remodeling and invasion in lung adenocarcinoma. Cancer Res. *84*, 1410–1425.
27. Butler, A., Hoffman, P., Smibert, P., Papalexi, E., and Satija, R. (2018). Integrating single-cell transcriptomic data across different conditions, technologies, and species. Nat. Biotechnol. *36*, 411–420.
28. Wu, T., Hu, E., Xu, S., Chen, M., Guo, P., Dai, Z., Feng, T., Zhou, L., Tang, W., Zhan, L., et al. (2021). clusterProfiler 4.0: a universal enrichment tool for interpreting omics data. Innovation *2*, 100141.
29. The Gene Ontology Consortium (2019). The Gene Ontology Resource: 20 years and still GOing strong. Nucleic Acids Res. *47*, D330–D338.

OMTON, Volume *34*

Supplemental information

AGTR1 signaling contributes to tumor immunity and therapy response in non-small cell lung cancer

Tatsuki Ikoma, Keigo Araki, Mai Kitagawa, Natsuno Makihara, Yutaro Nagata, Kazuki Fujii, Yukiko Okuno, Keisuke Kamisako, Yuta Okazaki, Kentaro Nakanishi, Yume Sanada, Kiyori Yoshida, Kahori Nakahama, Yuki Takeyasu, Utae Katsushima, Yuta Yamanaka, Satoshi Ikeda, Hiroshige Yoshioka, Toshio Shimizu, and Takayasu Kurata

Figure S1

(A) (B)

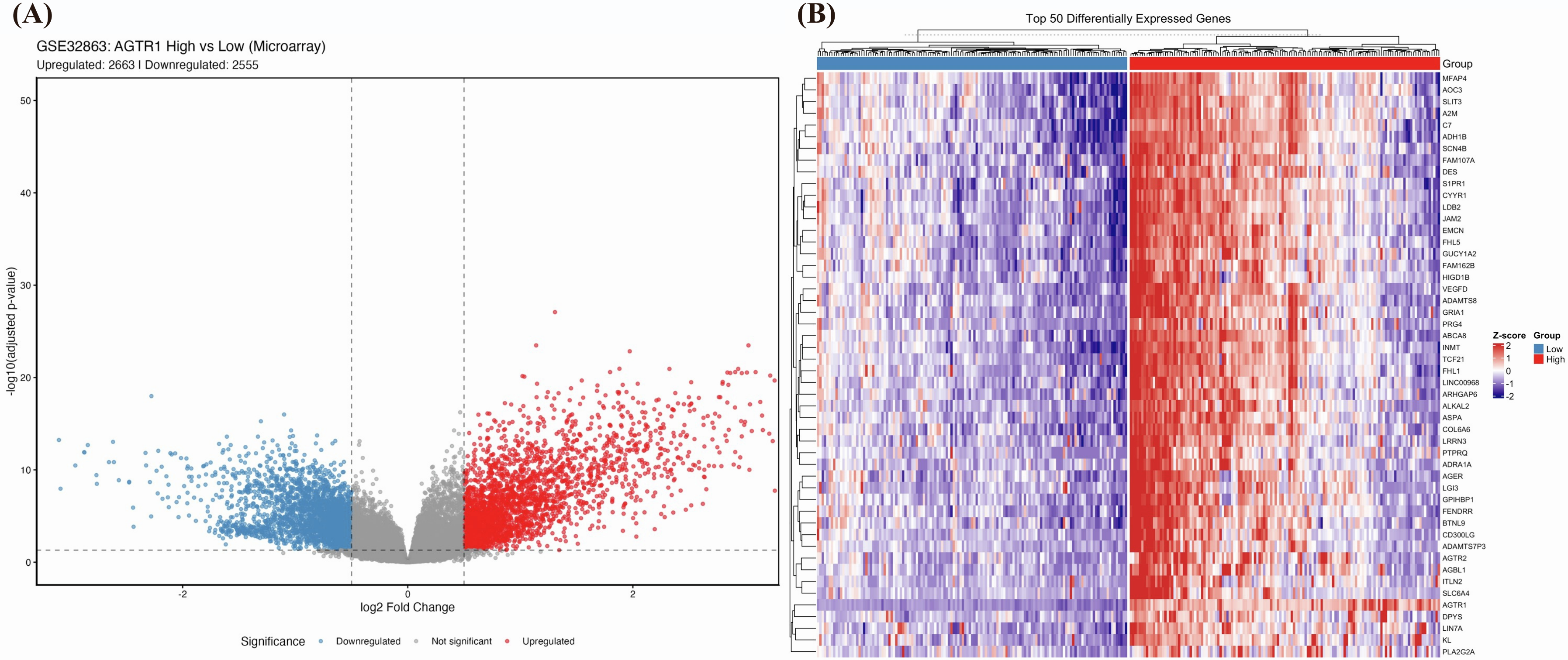
GSE32863: AGTR1 High vs Low (Microarray)
Upregulated: 2663 | Downregulated: 2555
-log10(adjusted p-value)
log2 Fold Change
Significance
Downregulated
Not significant
Upregulated
Top 50 Differentially Expressed Genes
Group
Z-score
Low
High
MFAP4
AOC3
SLIT3
A2M
C7
ADH1B
SCN4B
FAM107A
DES
S1PR1
CYYR1
LDB2
JAM2
EMCN
FHL5
GUCY1A2
FAM162B
HIGD1B
VEGFD
ADAMTS8
GRIA1
PRG4
ABCA8
INMT
TCF21
FHL1
LINC00968
ARHGAP6
ALKAL2
ASPA
COL6A6
LRRN3
PTPRQ
ADRA1A
AGER
LGI3
GPIHBP1
FENDRR
BTNL9
CD300LG
ADAMTS7P3
AGTR2
AGBL1
ITLN2
SLC6A4
AGTR1
DPYS
LIN7A
KL
PLA2G2A

(C)

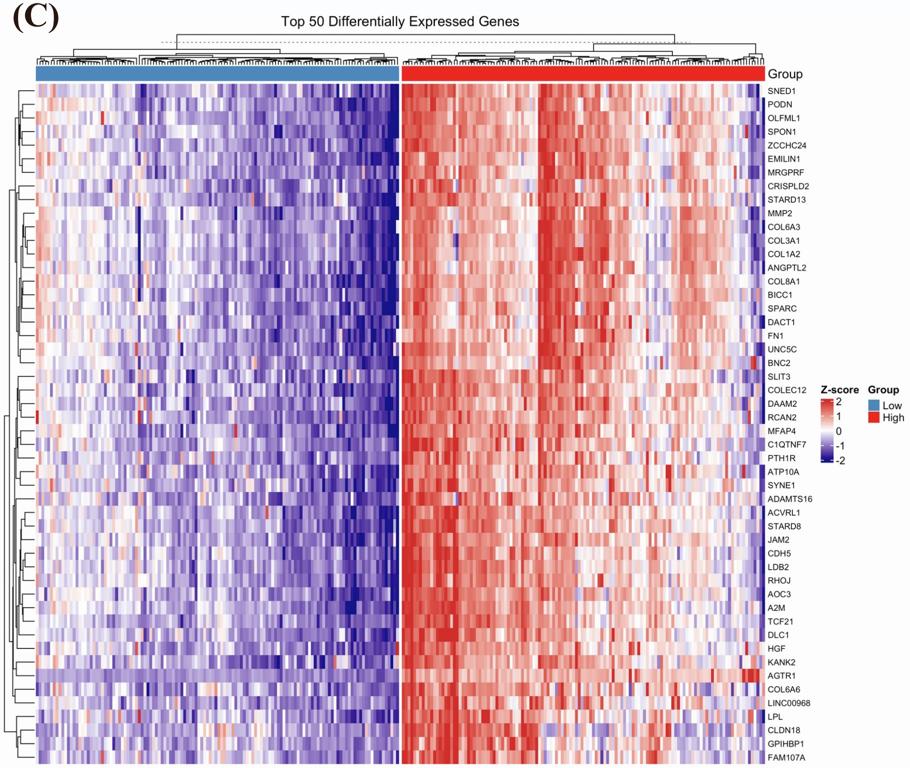

Top 50 Differentially Expressed Genes
Group
SNED1
PODN
OLFML1
SPON1
ZCCHC24
EMILIN1
MRGPRF
CRISPLD2
STARD13
MMP2
COL6A3
COL3A1
COL1A2
ANGPTL2
COL8A1
BICC1
SPARC
DACT1
FN1
UNC5C
BNC2
SLIT3
COLEC12
DAAM2
RCAN2
MFAP4
C1QTNF7
PTH1R
ATP10A
SYNE1
ADAMTS16
ACVRL1
STARD8
JAM2
CDH5
LDB2
RHOJ
AOC3
A2M
TCF21
DLC1
HGF
KANK2
AGTR1
COL6A6
LINC00968
LPL
CLDN18
GPIHBP1
FAM107A
Z-score
2
1
0
-1
-2
Group
Low
High

(D)

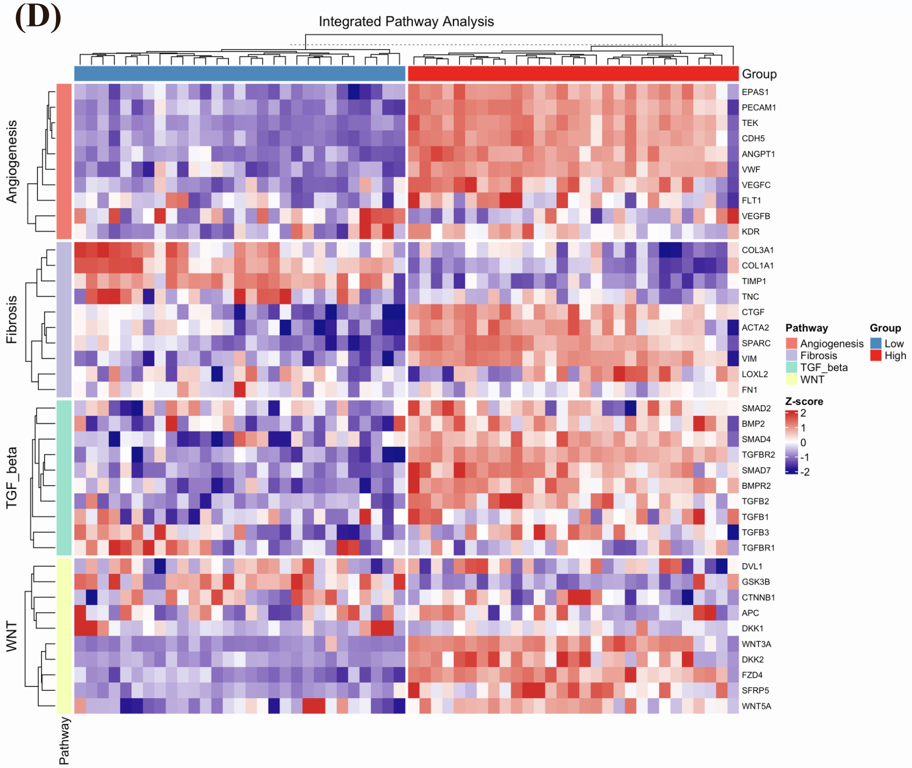

Integrated Pathway Analysis
Group
Angiogenesis
Fibrosis
TGF_beta
WNT
Pathway
EPAS1
PECAM1
TEK
CDH5
ANGPT1
VWF
VEGFC
FLT1
VEGFB
KDR
COL3A1
COL1A1
TIMP1
TNC
CTGF
ACTA2
SPARC
VIM
LOXL2
FN1
SMAD2
BMP2
SMAD4
TGFBR2
SMAD7
BMPR2
TGFB2
TGFB1
TGFB3
TGFBR1
DVL1
GSK3B
CTNNB1
APC
DKK1
WNT3A
DKK2
FZD4
SFRP5
WNT5A
Pathway
Angiogenesis
Fibrosis
TGF_beta
WNT
Group
Low
High
Z-score
2
1
0
-1
-2

(E)

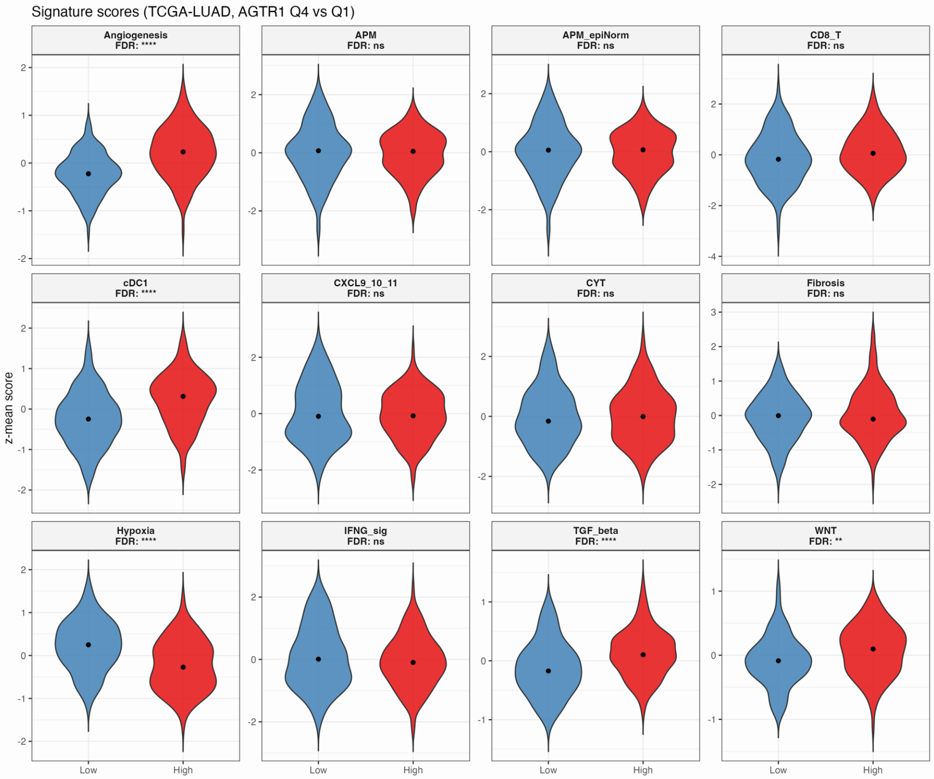
Signature scores (TCGA-LUAD, AGTR1 Q4 vs Q1)
Angiogenesis FDR: ****
APM FDR: ns
APM_epiNorm FDR: ns
CD8_T FDR: ns
cDC1 FDR: ****
CXCL9_10_11 FDR: ns
CYT FDR: ns
Fibrosis FDR: ns
Hypoxia FDR: ****
IFNG_sig FDR: ns
TGF_beta FDR: ****
WNT FDR: **
z-mean score
Low
High

(F)

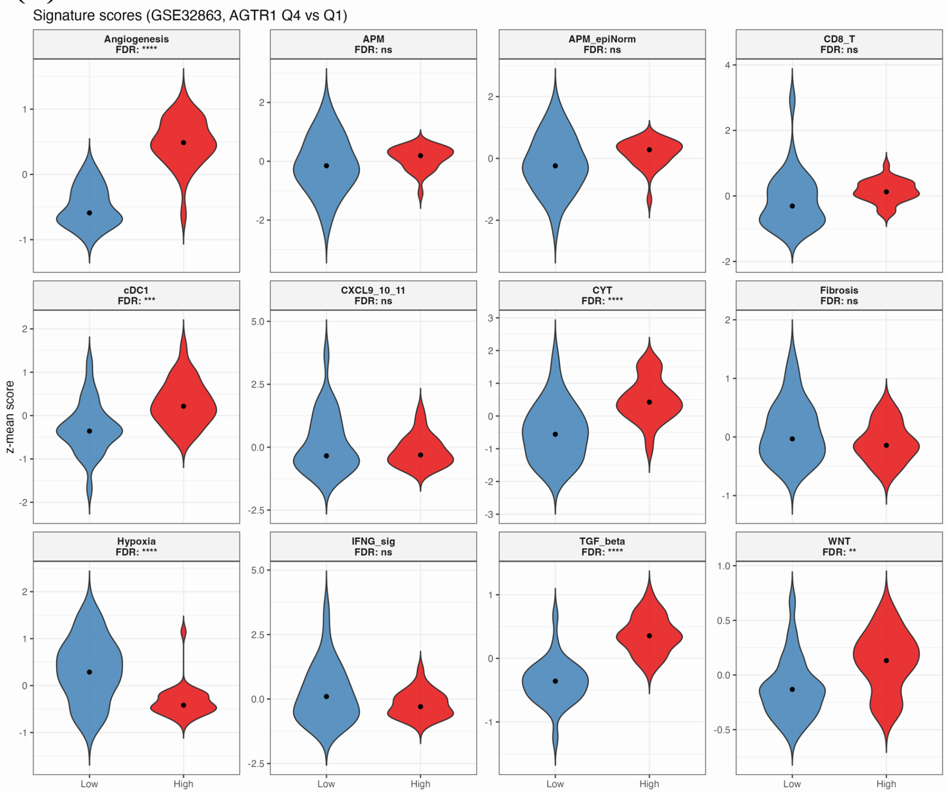
Signature scores (GSE32863, AGTR1 Q4 vs Q1)
Angiogenesis FDR: ****
APM FDR: ns
APM_epiNorm FDR: ns
CD8_T FDR: ns
cDC1 FDR: ***
CXCL9_10_11 FDR: ns
CYT FDR: ****
Fibrosis FDR: ns
Hypoxia FDR: ****
IFNG_sig FDR: ns
TGF_beta FDR: ****
WNT FDR: **
z-mean score
Low
High

(G)

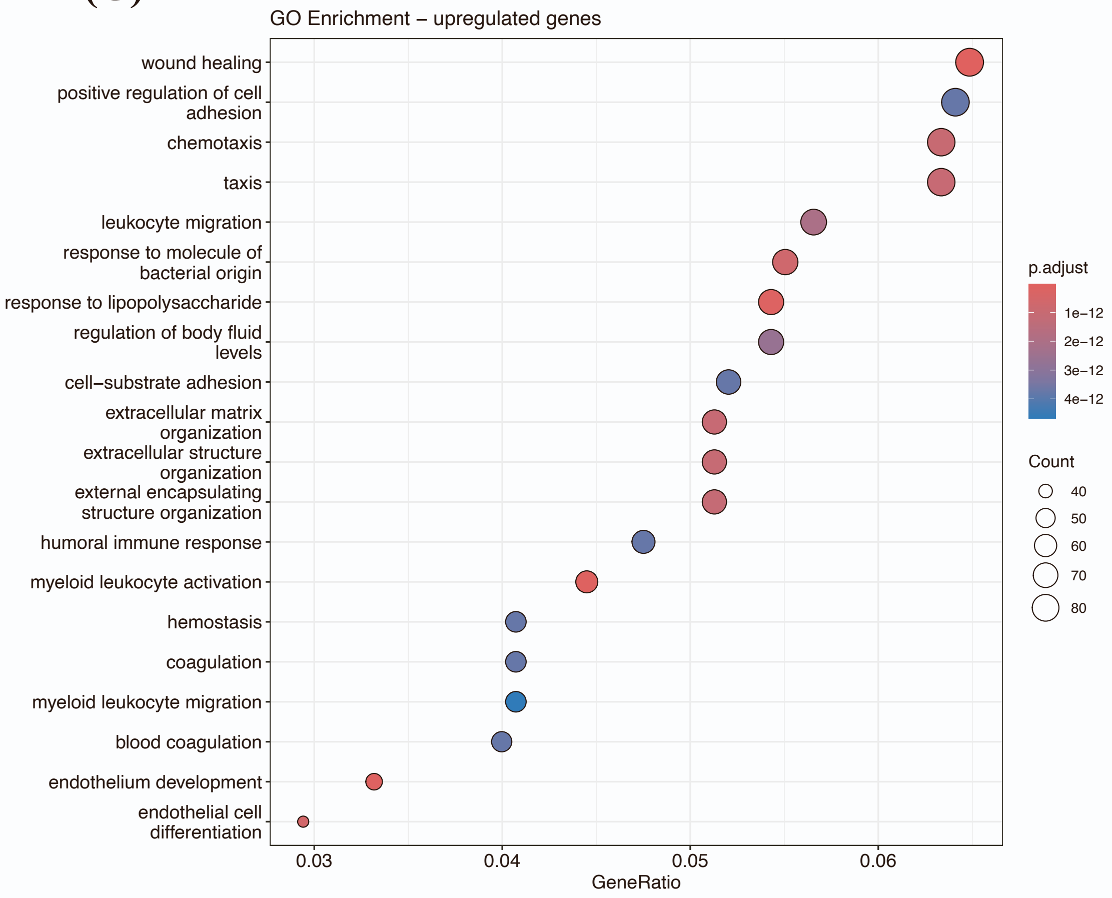
GO Enrichment – upregulated genes
wound healing
positive regulation of cell adhesion
chemotaxis
taxis
leukocyte migration
response to molecule of bacterial origin
response to lipopolysaccharide
regulation of body fluid levels
cell–substrate adhesion
extracellular matrix organization
extracellular structure organization
external encapsulating structure organization
humoral immune response
myeloid leukocyte activation
hemostasis
coagulation
myeloid leukocyte migration
blood coagulation
endothelium development
endothelial cell differentiation
0.03
0.04
0.05
0.06
GeneRatio
p.adjust
1e−12
2e−12
3e−12
4e−12
Count
40
50
60
70
80

Figure S1.
Additional validation analyses in the GSE32863 dataset and extended pathway assessments

Volcano plot showing differentially expressed genes between AGTR1-high and AGTR1-low groups in the GSE32863 dataset (Fig. A). Red dots indicate upregulated genes (n=2,663), blue dots indicate downregulated genes (n=2,555), and gray dots indicate non-significant genes. Vertical dashed lines represent log2 fold change thresholds, and the horizontal dashed line represents the adjusted p-value threshold. Heatmap showing the top 50 differentially expressed genes in the GSE32863 dataset (Fig. B). Samples are grouped by AGTR1 expression levels (low vs. high). The color scale represents Z-scores of gene expression, with red indicating high expression and blue indicating low expression. The other heatmap shows the top 50 differentially expressed genes, with hierarchical clustering, from the GSE32863 dataset (Fig. C). Notable genes include collagens, matrix metalloproteinases (MMP2), and various extracellular matrix-related genes. In Fig. D, the heatmap shows integrated pathway analysis of the GSE32863 dataset. Samples are grouped by AGTR1 expression. Four major pathway categories are shown: Angiogenesis, Fibrosis, TGF-beta, and WNT. The color scale represents pathway activity scores. Violin plots comparing pathway signature scores between AGTR1-low (blue) and AGTR1-high (red) groups in the TCGA-LUAD dataset, in Fig. E. Multiple pathway signatures are displayed, including Angiogenesis, APM, APM_epithelium, CD8 T cells, cDC1, CXCL9_10_11, CYT, Fibrosis, Hypoxia, IFNG_sig, TGF-beta, and WNT pathways. Statistical significance is denoted for significant comparisons. In Fig. F, the violin plots comparing pathway signature scores between AGTR1-low (blue) and AGTR1-high (red) groups in the GSE32863 dataset. The identical pathway signatures in panel E are analyzed for validation purposes. Note the differences in IFNG_sig and WNT pathway distributions compared to TCGA-LUAD. Bubble plot showing GO enrichment analysis of upregulated genes in the GSE32863 dataset. The x-axis represents GeneRatio, and the y-axis lists enriched biological processes. Bubble size indicates gene count, and color gradient represents adjusted p-value (p.adjust), with red indicating higher significance. Top enriched pathways include wound healing, chemotaxis, cell adhesion, leukocyte migration, and extracellular matrix organization.
